# Predictors of events of violence or aggression against nurses in the workplace: A scoping review

**DOI:** 10.1111/jonm.13635

**Published:** 2022-05-26

**Authors:** Nicola Pagnucci, Giulia Ottonello, Davide Capponi, Gianluca Catania, Milko Zanini, Giuseppe Aleo, Fiona Timmins, Loredana Sasso, Annamaria Bagnasco

**Affiliations:** ^1^ Department of Health Sciences University of Genoa Genoa; ^2^ School of Nursing, Midwifery & Health Systems University College Dublin Dublin Ireland

**Keywords:** consequences, nurse, nursing students, predictors, scoping review, workplace violence

## Abstract

**Aim:**

To identify predictors and consequences of violence or aggression events against nurses and nursing students in different work contexts.

**Background:**

Workplace violence against nurses and nursing students is a very common and widespread phenomenon. Actions to manage or prevent violent events could be implemented knowing the risk factors and consequences. However, there is a lack of systematic reviews that summarize knowledge on the predictors and consequences of workplace violence.

**Evaluation:**

A scoping review was conducted using electronic databases including APA PsycInfo, CINAHL, Cochrane, Ovid Medline, PubMed and Scopus.

**Key issues:**

After full text analysis, 87 papers were included in the current scoping review. Risk factors of horizontal violence were grouped into ‘personal’ and ‘Environmental and organizational’, and for violence perpetrated by patients into ‘personal’, ‘Environmental and organizational’ and ‘Characteristics of the perpetrators’.

**Conclusions:**

The results of this scoping review uncover problems that often remain unaddressed, especially where these episodes are very frequent. Workplace violence prevention and management programmes are essential to counter it.

**Implications for Nursing Management:**

The predictors and the consequents identified constitute the body of knowledge necessary for nurse managers to develop and implement policy and system actions to effectively manage or prevent violent events.

## 1 BACKGROUND

The International Labour Organization defines ‘workplace violence’ as ‘any action, incident or behaviour that departures from reasonable conduct in which a person is threatened, harmed, injured in the course of, or as a direct result of, his or her work’ (ILO‐International Labour Organization, 2003). The value of this definition lies both in its completeness (it covers all forms of violence), physical or psychological and in its inclusiveness (it does not exclude colleagues as a source of violence).

Health care professionals are often exposed to the risk of assault by patients or visitors. Workplace violence (WPV) among health care professionals, especially nurses, is the main occupational hazard in both developing and developed countries (Liu et al., 2019). A recent study reported that the prevalence of WPV against health care workers is high, especially in Asian and North American countries, psychiatric and emergency department settings, and among nurses and physicians (Liu et al., 2019).

More specifically, in North America, a survey conducted by the Emergency Nurses Association suggested that about one in every four nurses report having experienced physical violence more than 20 times in the previous 3 years and nearly a fifth report being verbally abused more than 200 times during the same period (Gacki‐Smith et al., 2009). The Australian Incident Monitoring System showed that out of a total of 42.33 accidents, 9% (*n* = 3621) involved health care professionals in events of violence perpetrated by patients, relatives or visitors (Benveniste et al., 2005). Recently, a large study conducted in Australia showed that more than 75% of the nurses and midwives suffered from violence perpetrated by patients and visitors in the previous six months (Pich & Roche, 2020).

A European study conducted in 2019 showed that out of 260 nurses from five different countries, 20.4%, confirmed they had been physically assaulted in the workplace in the previous 12 months and 76.9% of these reported that it was unavoidable; 92.3% reported being assaulted by patients, family members or visitors in their professional career (Babiarczyk et al., 2019). In particular, the emergency room has been identified as a high‐risk environment for WPV (Kowalenko et al., 2013), where nurses and trainees are the most exposed to this phenomenon (Chapman & Styles, 2006; Gerberich et al., 2005).

Although violent and aggressive patient behaviours are predominantly experienced by staff working in mental health units and emergency departments, patient violence and aggression are rising in other hospital areas, including general medicine and surgery units, paediatrics and intensive care (Ferri et al., 2016; Liu et al., 2019). Outside the hospital, episodes of violence and assaults have been suffered in‐home nursing services by 50% of nurses during their carriers (Fujimoto et al., 2017) and community care by 36% of nurses (Fafliora et al., 2016), as well as in pre‐hospital, ambulance and rescue services by 41% of nurses (Coskun Cenk, 2019; Velden et al., 2015).

Given the spread and the impact of this phenomenon, many studies have analysed the consequences of violence against nurses involving both physical and psychological consequences such as anger, fear or anxiety, post‐traumatic stress disorder symptoms (Hong et al., 2021), guilt, acute stress, decreased productivity (Al‐Ghabeesh & Qattom, 2019b), reduced job satisfaction (Berlanda et al., 2019), increased intention to leave, lower quality of life and even death (Çam & Ustuner Top, 2021; Heslop et al., 2019). The effects of violence in the health care setting may extend to the organization of the local service and entire health systems affecting the quality of services themselves. Health care organizations also incur in higher costs related to decreased productivity, poor job satisfaction and increased turnover (Speroni et al., 2014). Additional costs also result from lawsuits, compensation, and loss of revenue resulting from the negative image caused by violence incidents (Gerberich, 2004; Wax et al., 2016).

Although many health organizations around the world have implemented ‘zero tolerance’ policies for aggressors and established guidelines for the prevention and management of workplace violence, these policies often do not appear to work effectively in real life (Beattie et al., 2020; Hassankhani & Soheili, 2017; Morphet et al., 2014).

The most frequent risk factors of violence and aggression events include the characteristics of patients and nurses (e.g., gender, age and educational level) (Dangal et al., 2018; Zhu et al., 2021), weaknesses in leadership development or corporate policy implementation (Somani et al., 2021), poor training of personnel in the management of violence events (Jakobsson et al., 2021) and in recognizing risk situations, inadequate patient assessment and inadequate patient observation protocols (Palese et al., 2020), lack of communication between staff and patients, and their families (Yang et al., 2018) and deficiencies in the physical safety of the environment or in safety procedures (Babiarczyk et al., 2019; Najafi et al., 2018; Somani et al., 2021). All these factors and failure to recognize and respond to warning signals increase the risk of aggression or violence (Somani et al., 2021).

The identification of predictors or warning signals would enable health care professionals and managers to prevent and manage situations that could trigger events of violence in the workplace (Morphet et al., 2019). Furthermore, spreading the culture and knowledge of this phenomenon among health care professionals, managers and the general population could help to prevent the incidence of these episodes and protect both health care professionals and health service users.

## 2 OBJECTIVES

To identify predictors of violence or aggression against nurses and undergraduate nursing students in different health care settings.

Secondary objectives:

- Evaluate physical and psychosocial outcomes on nurses and undergraduate nursing students caused by violence or aggression and the economic and organizational consequences (unavailability and restoration of services).
- Describe episodes of violence or aggression against nurses and nursing students in the community setting.

Scoping review question

What are the predictors of the violence or aggression against nurses and students in different work contexts that enable their prevention or management?

Secondary questions:

What are the physical and psychosocial outcomes on nurses and nursing students of violence or aggression and the economic and organizational consequences?

Which violence or aggression events against nurses and nursing students in the community are described in the literature?

## 3 METHODS

### 3.1 Study design

The present review was developed according to the Joanna Briggs Institute (JBI) guidelines for scoping reviews (M. Peters, Godfrey, et al., 2020). The scoping review methodology was further refined, and corresponding guidance was developed by a working group from JBI and the JBI Collaboration (JBIC) (Aromataris & Munn, 2020; Peters et al., 2015).

A research question was developed based on the PEO components: Population (types of participants), Exposure of interest (independent variable) and Outcome (dependent variable).

The PRISMA‐ScR statement for scoping reviews (Tricco et al., 2018) was used to ensure the transparency of the study selection process.

The inclusion criteria are described in Table 1.

**TABLE 1:** Inclusion criteria

Type of participants	Exposure (independent variable)	Outcomes (dependent variable)	Types of studies
All studies, involving: Nurses Undergraduate nursing students working in any health setting.	All studies where predictors of violence or aggression against nurses and nursing students were identified or assessed with different tools. Predictive factors included, but not limited to external stimuli, such as institutional health systems and policies (often generating stressful situations) and work environment (structural, environmental and internal climate characteristics of work contexts) internal factors, such as intrinsic characteristics of patients, family members and other healthcare professionals (including but not limited to social status, personality disorders, past history of aggression, stress, substance and alcohol abuse, medical conditions, insecurity, attitude problems, sense of powerlessness, poor control, poor communication, frustration, anxiety and fear, different experience, skill levels and training).	Findings of violence or aggression against nurses or nursing students reported by the authors have been included in the review. The most interesting specific results were found in the studies including verbal abuse, psychological abuse, physical abuse, threats, intimidation, physical assaults, horizontal violence and various forms of bullying, in work‐related circumstances, carried out by users, family members or other healthcare professionals. The review included studies documenting outcomes on nurses and/or nursing students caused by physical violence or assault events (including but not limited to fractures, lacerations, bruises, sprains, back pain, bites or injuries, deprivation sleep, nausea and headache) emotional and psychological (including but not limited to stress, emotional exhaustion, burnout, anger, fear, loss of self‐esteem, loss of self‐confidence, anxiety, guilt, resentment, shock, embarrassment, humiliation, isolation and poor team cohesion) professional (including but not limited to lack of concentration, decreased job satisfaction, burnout, increased sick leave and decreased sensitivity to others) economic and/or organizational consequences caused by events of violence or aggression towards nurses or nursing students such as the reorganization of services, the implementation of time‐consuming activities to development of new policies and procedures, train and educate healthcare professionals, provide counselling services to victims, revise the organization due to turnover, sick leave and transfer of nurses to other departments, and temporary interruption or reduction of services offered to patients.	A wide range of study designs was considered appropriate to be as comprehensive as possible and to include the most significant number of studies for this review. Randomized controlled trials (RCTs) observational studies (e.g., prospective and retrospective cohort studies) case‐control studies cross‐sectional analytical studies Qualitative studies (e.g., phenomenological studies, ethnographic studies and Grounded Theory studies)

### 3.2 Search strategies

#### 3.2.1 Electronic databases

Based on the review question, six databases were searched: APA PsycInfo, Cumulative Index to Nursing and Allied Health Literature (CINAHL), Cochrane, Ovid Medline, PubMed and Scopus. Since no similar reviews were found, no time limit was set. Only papers in English and Italian were included.

The search terms were identified through the conceptual analysis conducted by Ventura‐Madangeng and Wilson (2009) and a further research of the literature.

The initial search strategy was as comprehensive as possible to include the largest number of studies, which were then gradually reduced based on the inclusion and exclusion criteria. Specific search strategies were adopted for each database. Table 2 shows the search concepts according to the PEO method and the keywords. The terms included synonyms or specific terms according to each database. The terms were combined as subject headings and text words in APA PsycInfo, CINAHL, Cochrane, Ovid Medline, PubMed and Scopus. The study selection process included two phases:

1. An initial screening of titles, abstracts and keywords according to the inclusion and exclusion criteria. The papers were independently selected by four reviewers. Studies were excluded even if only one inclusion criterion was not met. All duplicates were removed.
2. Full texts eligible for inclusion were read and analysed.

An external expert in scoping reviews supervised the entire selection and analysis process. All the papers were separately examined by two researchers and in case of disagreement a third researcher was involved to reach an agreement. The reasons for the exclusion of the full texts were recorded to track the decisions that were taken.

**TABLE 2:** Search concepts and keywords used (with appropriate Boolean operators)

Population: Nurse/Nurse student	Exposure: violence predictors	Outcome: Consequences of workplace violence	
Subject heading: In CINAHL: (‘nurses’ and ‘students, nursing’) In APA PsycInfo: (‘nurses’ and ‘nursing students’) In Medline: (‘nurses’ and ‘nursing students’) In PubMed, Cochrane: (‘Nurses’[Mesh] and ‘Students, Nursing’[Mesh])	Subject heading: In CINAHL: N/A In APA PsycInfo: N/A In Medline: N/A In PubMed, Cochrane: N/A	Subject heading: In CINAHL: (‘Workplace Violence’) In APA PsycInfo: (‘Workplace Violence’) In Medline: (‘Workplace Violence’) In PubMed, Cochrane: (‘Workplace Violence’[Mesh])	Subject heading: In CINAHL: (‘costs’) In APA PsycInfo: (‘costs’) In Medline: (‘Workplace Violence’) In PubMed, Cochrane: (‘Costs and Cost Analysis’[Mesh])
Keywords: nurse^a^ RN ‘registered nurse^a^’ ‘nursing student^a^’ ‘student nurse^a^’	Keywords: predictor^a^ Predicting antecedent^a^ ‘risk factor^a^’ ‘warning sign^a^’ ‘warning factor^a^’ ‘prediction sign^a^’ ‘prediction factor^a^’ ‘foreteller sing^a^’ ‘foreteller factor^a^’ foreshad^a^ forewarn^a^ sign^a^ factor^a^ harbinger^a^	Keywords:‘workplace violence’ aggression^a^ attack^a^ violence^a^ assault^a^ hostility abuse^a^ ‘physical aggression^a^’ ‘physical attack^a^’ ‘physical violence^a^’ ‘physical assault^a^’ ‘physical hostility’ ‘verbal aggression^a^’ ‘verbal attack^a^’ ‘verbal violence^a^’ ‘verbal abuse^a^’ ‘verbal assault^a^’ intimidation^a^ badgering bludgeoning deceive brainwash browbeat bulldoze bully^a^ ‘horizontal violence’ ‘lateral violence’ coerce constrain domineer harass intimidate oblige oppress persecute press push subjugate torment tyrannize	Keywords: cost^a^ ‘financial impact’ ‘financial burden’ ‘economic impact’ ‘financial cost^a^’ ‘economic cost^a^’ ‘monetary cost^a^’ ‘cost‐of‐illness’ ‘economic evaluation’ ‘illness cost^a^’ ‘medical cost^a^’ ‘health cost^a^’ ‘sick leave’ ‘turnover’ policies policy procedure^a^ ‘service interruption’ ‘reorganization of service’ ‘physical consequence^a^’ ‘physical injurie^a^’ ‘broken bone^a^’ laceration^a^ bruise^a^ sprain backache^a^ bite^a^ wound^a^ ‘sleep deprivation’ nausea headache^a^ pain ‘emotional consequence^a^’ ‘psychological consequence^a^’ disbelief ‘power’ ‘autonomy’ stress ‘emotional exhaustion’ depersonalization ‘personal accomplishment’ burnout anger fear ‘self‐esteem’ ‘self‐confidence’ anxiety ‘self‐blame’ resentment shock embarrassment humiliation isolation ‘team cohesion’

^a^ Any group of characters, including no character.

### 3.3 Data extraction

A data extraction sheet was developed according to the JBI guidelines for scoping reviews (M. Peters, Godfrey, et al., 2020).

The following data were collected:

Study design/methodology, purpose/objectives, research questions/hypotheses, study context (setting), sample description, sample size, exposure, tools for measuring results, results, methods of data analysis (statistical analysis), conclusions, comments and issues raised.

Data were extracted separately by two researchers.

### 3.4 Data synthesis

The results of the included studies underwent narrative synthesis, using words and text to summarize and explain the results. Its form varied from a simple account and description of the characteristics of the study, to the context, the quality and the results. Tables were used to compare the characteristics of the studies and the extracted data (Soilemezi & Linceviciute, 2018).

## 4 RESULTS

### 4.1 Selection of the studies included in the review

**FIGURE 1:**
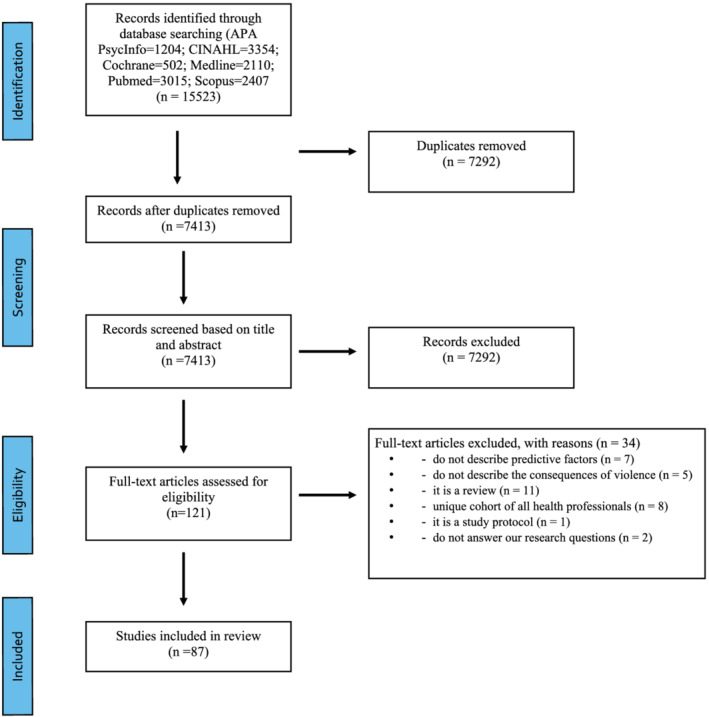
Flow diagram of the literature review process (PRISMA 2009)

A total of 15,523 records were initially identified after searching the databases. After titles and abstracts were screened, 121 papers underwent full text review. After reading the full texts, 87 papers were included in the current scoping review (see Figure 1, the PRISMA flow diagram).

### 4.2 Overview of the studies included in the review

Twenty‐eight studies were conducted in North America, 20 in Africa and the Middle East, 16 in Europe and 14 in Asia.

Of the studies included in the review, 59 analysed mainly the hospital setting and they involved all the departments (*n* = 31), the emergency room (*n* = 15), the psychiatric and mental health wards (*n* = 9), the operating room (*n* = 2), the medical and surgical departments (*n* = 1), the neonatal intensive care (*n* = 1) and in the trauma department (*n* = 1). Twenty‐four studies involved both hospital and community settings, of these 22 included various departments, and 2 were in mental health. Studies that exclusively analysed the community context, in the home care setting, were the least represented (*n* = 4). All the details regarding the context and sample characteristics of the included studies are shown in Table 3.

**TABLE 3:** Context and sample characteristics of included studies

Study reference	Country	Setting	Department	Sample description
Horizontal violence				
Al‐Ghabeesh and Qattom (2019b)	Jordan	Hospital	Emergency department	120 ED nurses
Anusiewicz et al. (2020)	USA	Hospital	Various departments	15 hospital nurses
Bambi et al. (2014)	Italy	Hospital	Emergency, intensive care, operating room departments	1202 ED, ICU, Operating Room nurses
Bambi et al. (2019)	Italy	Community and Hospital	Various departments	930 hospital and community nurses
Bardakçı and Günüşen (2014)	Turkey	Hospital	Various departments	284 hospital nurses
Blackstock et al. (2015)	Canada	Hospital	Various departments	103 hospital nurses
Bloom (2019)	USA	Hospital	Various departments	76 hospital nurses
Budin et al. (2013)	USA	Community and Hospital	Various departments	1407 hospital and community nurses
Chatziioannidis et al. (2018)	Greece	Hospital	Neonatal Intensive Care	233 neonatal intensive care nurses
Clarke et al. (2012)	Canada	Community and Hospital	Various departments	674 nursing students
Difazio et al. (2019)	Russia	Community and Hospital	Various departments	438 hospital and community nurses
		Community and Hospital	Various departments	998 community and hospital nurses
Favaro et al. (2021)	Canada	Community and Hospital	Various departments	1080 hospital and community nurses
Fontes et al. (2018)	Brazil	Community and Hospital	Various departments	419 hospital and community nurses
Hampton and Rayens (2019)	USA	Hospital	Various departments	170 nursing leaders
Hartin et al. (2020)	Australia	Community and Hospital	Various departments	70 hospital and community nurses
Higgins and MacIntosh (2010)	Canada	Hospital	Operating room	10 operating room nurses
Kozakova et al. (2018)	Czech Republic	Hospital	Various departments	456 hospital nurses
Laschinger and Grau (2012)	Canada	Community and Hospital	Mental Health	165 Psychiatric mental health nurses
Laschinger and Grau (2012)	Canada	Community and Hospital	Various departments	342 new graduate nurses
Laschinger et al. (2010)	Canada	Hospital	Various departments	415 hospital nurses
Park and Choi (2020)	South Korea	Hospital	Various departments	205 hospital nurses
Reknes et al. (2014)	Norway	Community and Hospital	Various departments	2059 hospital and community nurses
Serafin and Czarkowska‐Pączek (2019)	Poland	Community and Hospital	Various departments	404 hospital and community nurses
Yokoyama et al. (2016)	Japan	Community and Hospital	Various departments	825 hospital and community nurses
Violence perpetrated by patients and family members or visitors				
Avander et al. (2016)	Sweden	Hospital	Trauma Department	14 hospital nurses
Baby et al. (2014)	New Zealand	Community and Hospital	Mental Health	14 Psychiatric mental health nurses
Basfr et al. (2019)	Saudi Arabia	Hospital	Mental Health	310 Psychiatric mental health nurses
Bimenyimana et al. (2009)	South Africa	Hospital	Mental Health	10 Psychiatric mental health nurses
		Hospital	Various departments	592 hospital nurses
Boafo and Hancock (2017)	Ghana	Hospital	Various departments	92 hospital nurses
Estryn‐Behar et al. (2008)	Belgium, Germany, Finland, France, Italy, the Netherlands, Norway, Poland, Slovakia, UK	Community and Hospital	Various departments	39 898 hospital and community nurses
Evers et al. (2002)	The Netherlands	Community	Nursing Homes	551 community nurses
Farrell et al. (2014)	Australia	Community and Hospital	Various departments	1495 hospital and community nurses
Franz et al. (2010)	Germany	Community	Various departments	123 community nurses
Fujimoto et al. (2017)	Japan	Community	Mental Health	94 Psychiatric mental health nurses
Galián Muñoz et al. (2014)	Spain	Hospital	Emergency department	137 ED nurses
Gillespie et al. (2014)	USA	Hospital	Emergency department	177 ED nurses
Grainger and Whiteford (1993)	Australia	Hospital	Mental Health	717 incident report forms
Hahn et al. (2010)	Switzerland	Hospital	Various departments	291 hospital nurses
Hamdan and Hamra (2017)	Israel	Hospital	Emergency department	355 ED nurses
Hanohano (2017)	USA	Hospital	Mental Health	131 Psychiatric mental health nurses
Havaei et al. (2020)	Canada	Community and Hospital	Various departments	532 hospital and community nurses
Jenkins et al. (1998)	Ireland	Hospital	Emergency department	233 ED nurses
Jeong and Kim (2018)	South Korea	Hospital	Emergency department	246 ED nurses
Kobayashi et al. (2020)	Japan	Hospital	Mental Health	599 Psychiatric mental health nurses
Kowalenko et al. (2013)	USA	Hospital	Emergency department	117 ED nurses
Levin et al. (1998)	USA	Hospital	Emergency department	22 ED nurses
Ogundipe et al. (2013)	Nigeria	Hospital	Emergency department	81 ED nurses
Pinar and Ucmak (2011)	Turkey	Hospital	Various departments	255 hospital nurses
Ramacciati et al. (2019)	Italy	Hospital	Emergency department	816 ED nurses
Ray and Subich (1998)	USA	Hospital	Mental Health	78 Psychiatric mental health nurses
Rodney (2000)	Australia	Community	Nursing Homes	102 community nurses
Spelten et al. (2020)	Australia	Hospital	Emergency department	18 ED nurses
Speroni et al. (2014)	USA	Hospital	Various departments	762 hospital nurses
Tomagová et al. (2020)	Czech Republic	Hospital	Various departments	526 hospital nurses
Wolf et al. (2017)	USA	Hospital	Emergency department	16 ED nurses
Xing et al. (2015)	China	Hospital	Various departments	398 hospital nurses
Yang et al. (2018)	China	Hospital	Mental Health	290 hospital nurses
Zeng et al. (2013)	China	Hospital	Mental Health	387 Psychiatric mental health nurses
Both bullying and violence perpetrated by patients and family members or visitors				
Abou‐ElWafa et al. (2014)	Egypt	Hospital	Emergency and Medicine departments	134 ED nurses152 Internal medicine Department nurses
AbuAlRub et al. (2007)	Iraq	Hospital	Various departments	116 hospital nurses
AbuAlRub and Al‐Asmar (2011)	Jordan	Hospital	Various departments	422 hospital nurses
AbuAlRub and Al Khawaldeh (2014)	Jordan	Hospital	Various departments	396 hospital nurses
Aksakal et al. (2015)	Turkey	Hospital	Various departments	538 hospital nurses
Al‐Omari (2015)	Jordan	Hospital	Various departments	486 hospital nurses
Alameddine et al. (2015)	Lebanon	Hospital	Various departments	593 hospital nurses
Ceballos et al. (2020)	Brazil	Hospital	Emergency department	80 hospital nurses
Çelik and Çelik (2007)	Turkey	Community and Hospital	Various departments	622 hospital and community nurses
Cheung and Yip (2017)	Hong Kong	Hospital	Various departments	850 hospital nurses
Ferri et al. (2016)	Italy	Hospital	Various departments	125 hospital nurses
Hutton and Gates (2008)	USA	Hospital	Various departments	145 hospital nurses
Jafree (2017)	Pakistan	Hospital	Various departments	309 hospital nurses
Jaradat et al. (2016)	Palestine	Community and Hospital	Various departments	343 hospital and community nurses
Lash et al. (2006)	Turkey	Hospital	Various departments	73 nursing students
McKenna et al. (2003)	New Zealand	Community and Hospital	Various departments	551 hospital and community nurses
Merecz et al. (2020)	Poland	Community and Hospital	Various departments	413 hospital and community nurses
Nguluwe et al. (2016)	South Africa	Hospital	Mental Health	13 Psychiatric mental health nurses
Pai and Lee (2011)	Taiwan	Community and Hospital	Various departments	521 hospital and community nurses
Park et al. (2015)	South Korea	Hospital	Various departments	970 hospital nurses
Peters et al. (2020)	USA	Hospital	Various departments	279 hospital nurses
Read and Laschinger (2013)	Canada	Community and Hospital	Various departments	342 new graduate nurses
Sakellaropoulos et al. (2011)	USA	Hospital	Operating room	205 Nurse Anesthetists
Shi et al. (2017)	China	Hospital	Various departments	696 hospital nurses
Williams (1996)	USA	Community and Hospital	Various departments	345 hospital and community nurses
Wu et al. (2020)	China	Hospital	Various departments	1517 hospital nurses
Yang et al. (2012)	USA	Hospital	Various departments	176 hospital nurses

Regarding the designs of the included studies, the cross‐sectional descriptive design was adopted by 75 studies, 11 studies had a qualitative design and one a mixed‐methods design (Table 4).

**TABLE 4:** Designs of included studies (Total = 87)

Methodology	Number of articles	% of articles
**Quantitative**	**75**	**86.2**
Cross‐sectional descriptive design	74	98.6
Case report	1	0.4
**Qualitative**	**11**	12.6
Qualitative descriptive design	9	81.8
Qualitative phenomenological design	1	9.1
Qualitative historical design	1	9.1
**Mixed methods**	**1**	**1.2**
**Total**	**87**	**100**

The population mainly included nurses (85 studies), and nursing students (2 studies). All studies had higher percentages of female nurses or students (range = 58%–100%) except for the study by Xing. Nurses' work experience ranged between 1–23 years. The percentage of nurses with a diploma or (bachelor's) degree ranged between 38% and 93%. The Negative Act Questionnaire (NAQ) and the Workplace Violence in the Health Sector‐Country Case Study (WHO tool) to detect bullying and violent incidents were used in four studies.

### 4.3 Risk factors of violence

The forms of violence suffered by nurses and nursing students reported in the included studies are divided into *horizontal violence* perpetrated by professional co‐workers or by other students and clinical instructors (including different forms of bullying and mobbing), and *violence perpetrated by patients, family members, visitors or informal caregivers*. Table 5 shows in detail all the risk factors of WPV reported in the included studies.

**TABLE 5:** Risk factors of workplace violence reported in included studies

Risk factors of horizontal violence suffered by nurses		References
**Personal factors**		
Gender	Female	Anusiewicz et al. (2020) Ferri et al. (2016) Park et al. (2015) Sakellaropoulos et al. (2011) Serafin and Czarkowska‐Pączek (2019)
	Male	Chatziioannnidis et al. (2018) Difazio et al. (2019) Favaro et al. (2021) Jaradat et al. (2016) Nguluwe et al. (2016)
Age	Age 35 years old or younger	Jaradat et al. (2016)
	Age differences among nurses	Budin et al. (2013)
Educational level	College diploma	Favaro et al. (2021)
	Bachelor's degree	Bambi et al. (2019) Cheung and Yip (2017) Pai and Lee (2011)
	Master's degree	Hartin et al. (2020) Bardakçi and Günüşen (2014)
Work experience	<5 years (protective factor)	Bambi et al. (2014) Bardakçi and Günüşen (2014)
	Being young nurses	Bloom (2019) Favaro et al. (2021) Reknes et al. (2014)
	Less years of experience in current workplaces	Al‐Ghabeesh and Qattom (2019b) Chatziioannnidis et al. (2018) Higgins and MacIntosh (2010) Yokoyama et al. (2016)
**Environmental and organizational factors**		
Orientation of leadership towards situation or task Rigid hierarchical structures		Favaro et al. (2021) Fontes et al. (2018) Hampton and Rayens (2019) Laschinger et al. (2010) Laschinger and Grau (2012) Peters et al. (2020)
Low nurse manager ability		Bloom (2019) Fontes et al. (2018) Yokoyama et al. (2016)
Informal organizational alliances Tolerance of bullying		Blackstock et al. (2015)
Understaffing Increase in workload		AbuAlRub et al. (2007) AbuAlRub and Al‐Asmar (2011) Anusiewicz et al. (2020) Hartin et al. (2020) Kozakova et al. (2018) Serafin and Czarkowska‐Pączek (2019) Yokoyama et al. (2016)
High levels of stress		Bambi et al. (2019) Bloom (2019) Cheung and Yip (2017)
Unpredictability and constant change		Hartin et al. (2020)
Excessive competition between professionals		Hartin et al. (2020) Serafin and Czarkowska‐Pączek (2019)
Dayshift		Bambi et al. (2014) Bambi et al. (2019) Budin et al. (2013)
Nightshift		Park and Choi (2020) Reknes et al. (2014)
Structural empowerment (protective factor)		Favaro et al. (2021) Laschinger et al. (2010) Yokoyama et al. (2016)
Authentic leadership (protective factor)		Laschinger and Grau (2012) Read and Laschinger (2013) Yokoyama et al. (2016)

Risk factors of horizontal violence suffered by nursing students		References
**Personal factors**		
Gender	Female	Grainger and Whiteford (1993) Lash et al. (2006)
Age	Age 20 and 29 years old	Jafree (2017)
Marital status	Being single	Jafree (2017)
Religion	Being Muslim	Jafree (2017)
**Environmental and organizational factors**		
Morning shift		Grainger (1993) Jafree (2017)

Risk factors of violence suffered by nurses perpetrated by patients, family members or visitors		References
**Personal factors**		
Gender	Female	Ferri et al. (2016) Cheung and Yip (2017) Merecz et al. (2020) Boafo and Hancock (2017) Grainger and Whiteford (1993) Ramacciati et al. (2019) Tomagová et al. (2020) Xing et al. (2015)
	Female as protective factor	Al‐Omari (2015)
	Male	Alameddine et al. (2015) Jaradat et al. (2016) Yang et al. (2018) Zeng et al. (2013)
Age	Age 35 years old or younger	Boafo and Hancock (2017) Cheung and Yip (2017) Evers et al. (2002) Hahn et al. (2010) Kobayashi et al. (2020) Park et al. (2015) Sakellaropoulos et al. (2011) Yang et al. (2012)
	Age between 30 and 39 years old	Xing et al. (2015) Ramacciati et al. (2019)
	To be younger than one's patients	Nguluwe et al. (2016)
Work experience	<5 years	Al‐Omari (2015) Celik and Çelik (2007) Tomagová et al. (2020)
	>5 years	Ceballos et al. (2020) Fujimoto et al. (2017) Galián Muñoz et al. (2014) Hahn et al. (2010)
Educational level (Bachelor's degree or higher)		Cheung and Yip (2017) Hahn et al. (2010) Kowalenko et al. (2013) Zeng et al. (2013)
Negative personal attitudes and behaviours		Hamdan and Hamra (2017) Christopher (1998) Wolf et al. (2017)
Poor communication skills		AbuAlRub and Al Khawaldeh (2014) Nguluwe et al. (2016) Yang et al. (2018)
**Environmental and organizational factors**		
Understaffing		Basfr et al. (2019) Bimenyimana et al. (2009) Grainger and Whiteford (1993) Ogundipe et al. (2013) Yang et al. (2018)
Working department	Work in emergency departments	Estryn‐Behar et al. (2008) Farrell et al. (2014) Ferri et al. (2016) Hahn et al. (2010) Jenkins et al. (1998) Jeong and Kim (2018) Pinar and Ucmak (2011) Ramacciati et al. (2019) Speroni et al. (2014) Tomagová et al. (2020) Williams (1996)
	Work in psychiatric settings	Estryn‐Behar et al. (2008) Farrell et al. (2014) Ferri et al. (2016) Franz et al. (2010) Yang et al. (2018)
	Work in geriatric settings	Estryn‐Behar et al. (2008) Farrell et al. (2014) Ferri et al. (2016) Hahn et al. (2010)
	Work in nursing homes and long‐term care	Franz et al. (2010) Williams (1996)
Working in shifts		Abou‐ElWafa et al. (2014) Aksakal et al. (2015) Alameddine et al. (2015) Basfr et al. (2019) Ceballos et al. (2020) Cheung and Yip (2017) Estryn‐Behar et al. (2008) Farrell et al. (2014) Ferri et al. (2016) Grainger and Whiteford (1993) Hanohano (2017) Pai and Lee (2011) Yang et al. (2018) Zeng et al. (2013)
High workload Time pressure Physical load		Estryn‐Behar et al. (2008) Evers et al. (2002) Hanohano (2017) Jafree (2017) Yang et al. (2012)
Low quality of physical working environment		Havaei et al. (2020) Wu et al. (2020)
Providing direct patient care Being a front‐line nurse		Cheung and Yip (2017) Gillespie et al. (2014) Hahn et al. (2010) Hutton and Gates (2008) Speroni et al. (2014) Xing et al. (2015)
Long waiting time		Basfr et al. (2019) Gillespie et al. (2014) Hamdan and Hamra (2017) Levin et al. (1998) Kowalenko et al. (2013) Ogundipe et al. (2013) Yang et al. (2018)
Unmet expectations of patients/families		Basfr et al. (2019) Hamdan and Hamra (2017) Ogundipe et al. (2013) Yang et al. (2018)
Lack of anti‐violence policies Lack of procedures to report WPV		AbuAlRub et al. (2007) AbuAlRub and Al Khawaldeh (2014) Alameddine et al. (2015) Gillespie et al. (2014) Xing et al. (2015)
Inadequate security system		Jenkins et al. (1998) Levin et al. (1998) Merecz et al. (2020) Ogundipe et al. (2013)
**Characteristics of the perpetrators**		
Patient alcohol or drug abuse		Avander et al. (2016) Baby et al. (2014) Ferri et al. (2016) Hamdan and Hamra (2017) Nguluwe et al. (2016) Ogundipe et al. (2013) Spelten et al. (2020) Speroni et al. (2014)
Mental status and patient conditions		Baby et al. (2014) Bimenyimana et al. (2009) Levin et al. (1998) Spelten et al. (2020) Yang et al. (2018) Cheung and Yip (2017)
Dementia or Alzheimer		Speroni et al. (2014) Nguluwe et al. (2016)
Pain		Hamdan and Hamra (2017)
Anxiety Fear		Hamdan and Hamra (2017) Christopher (1998) Pai and Lee (2011) Shi et al. (2017)
Patients with aggressive behaviours		AbuAlRub and Al‐Asmar (2011) Rodney (2000)
Patients with unrealistic expectations		Gillespie et al. (2014) Hamdan and Hamra (2017) Pai and Lee (2011) Speroni et al. (2014)

Risk factors of violence suffered by nursing students perpetrated by patients, family members or visitors		References
**Personal factors**		
Gender	Female	Grainger and Whiteford (1993) Lash et al. (2006)
Age	Age between 20 and 29 years	Jafree (2017)
Marital status	Being single	Jafree (2017)
**Environmental and organizational factors**		
Being the least knowledgeable and least powerful group		Lash et al. (2006)
During patient refusing a request		Grainger (1993)
Placement in a psychiatric ward		Grainger (1993)
Placement in emergency room		Jafree (2017)
**Characteristics of the perpetrators**		
Inexperienced clinical instructors		Lash et al. (2006)
Patients with aggressive behaviours		Grainger (1993) Jafree (2017)

#### 4.3.1 Risk factors of horizontal violence suffered by nurses

Horizontal violence factors can be divided into personal and environmental/organizational factors.

##### Personal factors

Contrasting findings were reported with regard to nurses' gender; in some studies ‘being a male nurse’ was reported as a predictor (Chatziioannidis et al., 2018; Jaradat et al., 2016), whereas in others, ‘being a female nurse’ was considered a predictor (Anusiewicz et al., 2020; Park et al., 2015). In addition, being a young nurse (Bloom, 2019; Favaro et al., 2021; Reknes et al., 2014) or having few years of experience in the current workplace (Al‐Ghabeesh & Qattom, 2019b; Chatziioannidis et al., 2018; Higgins & MacIntosh, 2010; Yokoyama et al., 2016) were described as factors related to the risk of being bullied. On the contrary, other authors found that a work experience of <5 years was a factor that protected nurses from horizontal violence (Bambi et al., 2019; Bardakçı & Günüşen, 2014).

##### Environmental and organizational factors

These factors included situation‐ or task‐oriented leadership, rigid hierarchical structures (Favaro et al., 2021; Fontes et al., 2018; Hampton & Rayens, 2019; Laschinger & Grau, 2012; Laschinger et al., 2010; A. Peters, El‐Ghaziri, et al., 2020), informal organizational alliances (i.e., covert coalitions of bullies) and the consequent abuse of organizational procedures (Blackstock et al., 2015). Furthermore, several studies identified the increase in workload and understaffing, pressure placed on workers (AbuAlRub et al., 2007; AbuAlRub & Al‐Asmar, 2011; Anusiewicz et al., 2020; Hartin et al., 2020; Kozakova et al., 2018; Serafin & Czarkowska‐Pączek, 2019; Yokoyama et al., 2016) and high levels of stress (Bambi et al., 2019; Bloom, 2019; Cheung & Yip, 2017) as factors facilitating mobbing or bullying. Numerous authors identified structural empowerment and authentic leadership as protective factors against bullying in the workplace with a statistically significant negative correlation between these variables (Favaro et al., 2021; Laschinger et al., 2010; Laschinger & Grau, 2012; Read & Laschinger, 2013; Yokoyama et al., 2016).

#### 4.3.2 Risk factors of horizontal violence suffered by nursing students

##### Personal factors

‘Being female’ is reported as a predictor of bullying for nursing students by Grainger and Whiteford (1993) and Lash et al. (2006). According to Jafree (2017), having an age between 20 and 29 years, single marital status, and following the Muslim religion are predictors of horizontal violence.

##### Environmental and organizational factors

Attending clinical internship during the day shifts is reported as a predictor of horizontal violence for students by Grainger and Whiteford (1993) and Jafree (2017).

#### 4.3.3 Risk factors of violence suffered by nurses perpetrated by patients, family members or visitors

These include personal factors, environmental/organizational factors and characteristics of aggressors.

##### Personal factors

Gender of health workers is controversially identified as a factor that increases the risk of suffering violence. In some studies, ‘male gender’ was associated with a higher risk of suffering WPV (Alameddine et al., 2015; Jaradat et al., 2016; Yang et al., 2018; Zeng et al., 2013), while, according to other studies, this risk was associated with ‘female gender’ (Boafo & Hancock, 2017; Cheung & Yip, 2017; Ferri et al., 2016; Grainger & Whiteford, 1993; Merecz et al., 2020; Ramacciati et al., 2019; Tomagová et al., 2020; Xing et al., 2015). Instead, according to Al‐Omari (2015), being a female protects from violence. Another factor is younger age. Several studies found that those aged <35 years were most at risk (Boafo & Hancock, 2017; Cheung & Yip, 2017; Evers et al., 2002; Hahn et al., 2010; Kobayashi et al., 2020; Park & Choi, 2020; Sakellaropoulos et al., 2011; Yang et al., 2012). In particular, being younger than one's patients was another factor that increases the risk of suffering violence (Nguluwe et al., 2016). Other authors identified the 30‐to 39‐year age group (Ramacciati et al., 2019; Xing et al., 2015) as the one most at risk. Also, having a bachelor's degree or higher educational level has identified as predictor of WPV (Cheung & Yip, 2017; Hahn et al., 2010; Kowalenko et al., 2013; Zeng et al., 2013).

Work experience was also identified as a predictor of WPV. Controversially, some authors found that having <5 years of service increased the risk of suffering violence (Al‐Omari, 2015; Çelik & Çelik, 2007; Tomagová et al., 2020), while for others this was higher in those with a career of >5 years (Ceballos et al., 2020; Fujimoto et al., 2017; Galián Muñoz et al., 2014; Hahn et al., 2010).

##### Environmental and organizational factors

Many studies have identified several departments as risk factors for WPV. Working in emergency departments (Estryn‐Behar et al., 2008; Farrell et al., 2014; Ferri et al., 2016; Hahn et al., 2010; Jenkins et al., 1998; Jeong & Kim, 2018; Pinar & Ucmak, 2011; Ramacciati et al., 2019; Speroni et al., 2014; Tomagová et al., 2020; Williams, 1996), psychiatric settings (Estryn‐Behar et al., 2008; Farrell et al., 2014; Ferri et al., 2016; Franz et al., 2010; Yang et al., 2018), geriatric settings (Estryn‐Behar et al., 2008; Farrell et al., 2014; Ferri et al., 2016; Hahn et al., 2010) or in nursing homes and long‐term care (Franz et al., 2010; Williams, 1996) expose nurses to a greater risk of violence. Various working organizational aspects and having scarce resources are identified as risk factors for WPV: inadequate staffing levels (Basfr et al., 2019; Bimenyimana et al., 2009; Grainger & Whiteford, 1993; Ogundipe et al., 2013; Yang et al., 2018), high workload, time pressure and physical load (Estryn‐Behar et al., 2008; Evers et al., 2002; Hanohano, 2017; Jafree, 2017; Yang et al., 2012). The type of job contract is another predisposing factor. Working full‐time and in shifts was associated with a higher risk of violence (Abou‐ElWafa et al., 2014; Aksakal et al., 2015; Alameddine et al., 2015; Basfr et al., 2019; Ceballos et al., 2020; Cheung & Yip, 2017; Estryn‐Behar et al., 2008; Farrell et al., 2014; Ferri et al., 2016; Grainger & Whiteford, 1993; Hanohano, 2017; Pai & Lee, 2011; Yang et al., 2018; Zeng et al., 2013). Another predisposing factor of violence is long waiting times for patients, especially in the emergency department (Basfr et al., 2019; Gillespie et al., 2014; Hamdan & Hamra, 2017; Kowalenko et al., 2013; Levin et al., 1998; Ogundipe et al., 2013; Yang et al., 2018).

##### Characteristics of violence perpetrators

Nurses caring for patients suffering from psychiatric disorders or advanced dementias both in the community and in the hospital, report higher rates of physical and verbal violence (Nguluwe et al., 2016; Speroni et al., 2014). Alcohol or drug abuse by patients (Avander et al., 2016; Baby et al., 2014; Ferri et al., 2016; Hamdan & Hamra, 2017; Nguluwe et al., 2016; Ogundipe et al., 2013; Spelten et al., 2020; Speroni et al., 2014) and their mental status and clinical conditions (Baby et al., 2014; Bimenyimana et al., 2009; Cheung & Yip, 2017; Levin et al., 1998; Spelten et al., 2020; Yang et al., 2018), as well as aggressive patients' behaviors (AbuAlRub & Al‐Asmar, 2011; Rodney, 2000), expose nurses to a higher risk of violence.

#### 4.3.4 Risk factors of violence suffered by nursing students perpetrated by patients, family members or visitors

##### Personal factors

‘Being female’ (Grainger & Whiteford, 1993; Lash et al., 2006), having an age range of 20–29 years and being single (Jafree, 2017) increase the risk of suffering violence among nursing students.

##### Environmental and organizational factors

Being in the least knowledgeable and with the least decisional power (Lash et al., 2006) together with being present when a patient refuses a request (Grainger & Whiteford, 1993) are seen as environmental and organizational predictors of violence. Also, internships in psychiatric wards (Grainger & Whiteford, 1993) or the emergency room (Jafree, 2017) are other risk factors.

##### Characteristics of perpetrators

Usually, the perpetrators of violence towards nursing students are either inexperienced clinical instructors (Lash et al., 2006) or patients with aggressive behaviours (Grainger & Whiteford, 1993; Jafree, 2017).

### 4.4 Consequences of violence

The consequences of workplace violence suffered by nurses and nursing students reported in the included studies are divided into ‘*Professional life’* and ‘*Emotional and psychological wellbeing’* for horizontal violence, together with ‘*Physical consequences’ and ‘Consequences for the work environment, damage and costs’* for violence perpetrated by patients and visitors. Table 6 shows details of WPV consequences.

**TABLE 6:** Consequences of workplace violence reported in included studies

Consequences of horizontal violence suffered by nurses	References
**Professional life**	
Professional life	Al‐Ghabeesh and Qattom (2019b)
Quality of care provided Less adequate responses and low patient safety	Çelik and Çelik (2007)
Greater precariousness of work and with less control on clinical practices	Park and Choi (2020)
Poor overall job satisfaction	Hartin et al. (2020)
**Emotional and psychological wellbeing**	
Increased impulsiveness Anxiety Depression	Bambi et al. (2014) Blackstock et al. (2015) Favaro et al. (2021) Fontes et al. (2018) Kozakova et al. (2018)
Sadness Burnout Emotional exhaustion	Laschinger et al. (2010)
Sense of guilt	Bambi et al. (2014)
Feeling victims	Chatziioannidis et al. (2018)
Cynicism	Laschinger et al. (2010)
Chronic fatigue Low concentration Sleep disturbances	Bambi et al. (2014) Difazio et al. (2019)

Consequences of horizontal violence suffered by nursing students	References
**Professional life**	
Intentions to leave the nursing programme	Clarke et al. (2012)
Increased rates of absenteeism from internship placement	Lash et al. (2006)
Becoming super alert or watchful and on guard	Clarke et al. (2012)
**Emotional and psychological wellbeing**	
Loss of appetite Difficulty falling asleep Headache Tiredness	Lash et al. (2006)

Consequences of violence suffered by nurses perpetrated by patients, family members or visitors	References
**Professional life**	
Poor job satisfaction	AbuAlRub and Al Khawaldeh (2014) AbuAlRub and Al‐Asmar (2011) Al‐Omari (2015) Galian‐Munoz et al. (2014) Jaradat et al. (2016) Kobayashi et al. (2020) Merecz et al. (2020)
Loss of interest in work	Bimenyimana et al. (2009)
Increased fear of new episodes of violence at work	Park et al. (2015) Ramacciati et al. (2019)
Constant state of alertness	Al‐Omari (2015) Jafree (2017) Tomagová et al. (2020)
Sense of diminished security	Ogundipe et al. (2013)
Reducing to the minimum their contacts with patients and the time spent to treat patients	Hamdan and Hamra (2017)
Increased absence from work	AbuAlRub and Al Khawaldeh (2014) Jenkins et al. (1998) Speroni et al. (2014) Xing et al. (2015)
Increased intention to change the workplace and leave the profession	Bimenyimana et al. (2009) Hutton and Gates (2008) Ogundipe et al. (2013) Pinar and Ucmak (2011)
**Emotional and psychological wellbeing**	
Anxiety Fear Frustration	Farrell et al. (2014) Basfr et al. (2019) McKenna et al. (2003) Nguluwe et al. (2016) Pinar and Ucmak (2011)
Depression Anger Cynicism Nervousness Helplessness	Galian‐Munoz et al. (2014) Nguluwe et al. (2016) Yang et al. (2018) Bimenyimana et al. (2009)
Depersonalization Stress Burnout Emotional exhaustion	Wu et al. (2020) Yang et al. (2018) Bimenyimana et al. (2009) Franz et al. (2010)
Recurring memories and thoughts related to violence Nightmares Sleep disturbances	Bambi et al. (2019) Levin et al. (1998) Ogundipe et al. (2013)
Loss of appetite Gastrointestinal disorders	Bambi et al. (2019)
Drug abuse by nurses and alcoholism	Bimenyimana et al. (2009)
High risk of post‐traumatic stress disorder (PTSD) Loss of self‐confidence	McKenna et al. (2003)
**Physical consequences**	
Scratches Bruises Abrasions Swelling Muscle tension	Yang et al. (2018)
Fractures Musculoskeletal injuries Head injuries Asphyxia Lacerations Sensory deficits Physical disability Chronic pain	Baby et al. (2014) Levin et al. (1998) Nguluwe et al. (2016) Yang et al. (2018)
One to five days of sick leave Medical examination Specific treatment	Franz et al. (2010) Speroni et al. (2014)
Death	AbuAlRub et al. (2007)
**Consequences for the work environment, damages and costs**	
Damage to the furniture	Gillespie et al. (2014)
Damage tools and structures of health care facilities	Galian‐Munoz et al. (2014)
Loss of a regular salary Cost of medical care and long‐term leave from work for the recovery process	Favaro et al. (2021)
Long‐term health care Rehabilitation Victim reintegration Unemployment Retraining costs for victims who lose or leave their jobs Injury benefits and treatment costs	Favaro et al. (2021) Speroni et al. (2014)
Increasing staff turnover with a cost of up to $ 337,500 Inability to hire due to turnover costs	AbuAlRub et al. (2007)
Decrease in productivity	Hutton and Gates (2008)

Consequences of violence suffered by nursing students perpetrated by patients, family members or visitors	References
**Professional life**	
Increased rates of absenteeism from internship placement	Clarke et al. (2012)
**Emotional and psychological wellbeing**	
Disturbing memories Thoughts Images of the attack	Clarke et al. (2012)
Sense of helplessness Loss of self‐esteem Neglect of self‐care	Lash et al. (2006)

#### 4.4.1 Consequences of horizontal violence suffered by professional nurses

##### Professional life

The most frequent consequence is the increasing intention to change workplace or to leave the nursing profession (Bambi et al., 2014; Blackstock et al., 2015; Favaro et al., 2021; Fontes et al., 2018; Kozakova et al., 2018).

##### Emotional and psychological wellbeing

At the same time the increasing of impulsiveness, anxiety and depression is the most frequent emotional and psychological consequence (Bambi et al., 2014; Blackstock et al., 2015; Favaro et al., 2021; Fontes et al., 2018).

#### 4.4.2 Consequences of horizontal violence suffered by nursing students

##### Professional life

The most frequent consequences for nursing students of horizontal violence are the intention to leave the nursing programme (Clarke et al., 2012), the increased rates of absenteeism from internship placement (Lash et al., 2006).

##### Emotional and psychological wellbeing

The most frequently reported physical and emotional consequences are headache, loss of appetite and difficulty falling asleep (Lash et al., 2006).

#### 4.4.3 Consequences of violence suffered by nurses perpetrated by patients, family members or visitors

##### Professional life

The most frequent consequences suffered by professional nurses of violence perpetrated by patients are poor job satisfaction (AbuAlRub & Al Khawaldeh, 2014; AbuAlRub & Al‐Asmar, 2011; Al‐Omari, 2015; Galián Muñoz et al., 2014; Jaradat et al., 2016; Kobayashi et al., 2020; Merecz et al., 2020), increased absence from work (AbuAlRub & Al Khawaldeh, 2014; Jenkins et al., 1998; Speroni et al., 2014; Xing et al., 2015) and increased intention to change workplace and leave the profession (Bimenyimana et al., 2009; Hutton & Gates, 2008; Ogundipe et al., 2013; Pinar & Ucmak, 2011).

##### Emotional and psychological wellbeing

Stress and burnout due to violence perpetrated by patients, family members or visitors (Bimenyimana et al., 2009; Franz et al., 2010; Gillespie et al., 2014; Wu et al., 2020; Yang et al., 2018).

##### Physical consequences

The most common physical consequences are lacerations, musculoskeletal injuries, fractures, physical disability, chronic pain and head injuries (Baby et al., 2014; Levin et al., 1998; Nguluwe et al., 2016; Yang et al., 2018).

##### Consequences for the work environment, damage and costs

The consequences for the workplace environment range from damage to the furniture (Gillespie et al., 2014), tools and structures of health care facilities (Galian‐Munoz et al., 2014). The physical consequences of the violent events also have economic repercussions in terms of loss of regular salary, cost of medical care and long‐term leave from work for the recovery process (Favaro et al., 2021). WPV episodes increase staff turnover with a cost of up to $ 337,500; this leads to inability to hire, generating a toxic work environment and a lack of loyalty and cooperation (AbuAlRub et al., 2007). In a study conducted in the USA, the decrease in productivity was approximately $ 1300 for each nurse that experienced violence (Hutton & Gates, 2008).

#### 4.4.4 Consequences of violence suffered by nursing students perpetrated by patients, family members or visitors

##### Professional life

Consequences for students due to violence perpetrated by patients reported by the studies included in our review involve the increasing rates of absenteeism from internship placements (Clarke et al., 2012).

##### Emotional and psychological wellbeing

Studies reveal disturbing memories and negative thoughts (Clarke et al., 2012), loss of self‐esteem and sense of helplessness (Lash et al., 2006) as the main emotional and psychological consequences for nursing students.

## 5 DISCUSSION

The phenomenon of workplace violence is widespread and documented worldwide. The literature describes violence mainly in hospital settings and in emergency rooms but also in community services and in various hospital departments. The present review enabled to identify several risk factors of WPV.

### 5.1 Risk factors and consequences of horizontal violence

Horizontal violence is facilitated by specific personal factors of victims such as gender, age educational level and work experience. A way to promote integration and respect among professionals and prevent horizontal violence could be creating teams of nurses that have a good balance in terms of gender, age, a mix of work experience and skills to achieve common goals and greater autonomy (Edmonson & Zelonka, 2019).

Several environmental and organizational factors, such as poor nurse manager skills, rigid and hierarchical structures, understaffing, high levels of stress, shift work and unhealthy competition between professionals have been reported as additional risk factors for horizontal violence. The replacement of the current situation‐oriented or task‐oriented leadership with structural empowerment processes (Goedhart et al., 2017) aimed at achieving goals through access to information, support, resources and opportunities (Moura et al., 2020) can reduce bullying and mobbing. Furthermore, constant organizational changes and staff shortages increase nurses' stress levels. High levels of stress and job dissatisfaction, as well as leading to adverse patient outcomes (Bloom, 2019; Brooks Carthon et al., 2021; Schlak et al., 2021), create a favourable substrate for horizontal violence.

Nursing students suffer from WPV, too. Likewise, the students' personal factors such as gender, age, marital status and religion have been identified as risk factors of horizontal violence. In order to prevent the bullying of students, faculty members should acknowledge the inherent vulnerability of learners, their personal risk factors and also reflect on their own communication practices and how these impact on learners (Seibel & Fehr, 2018).

### 5.2 Risk factors and consequences of violence perpetrated by patients or family members

In many studies included in this review, victims' personal characteristics such as gender, age, work experience and educational level, are reported to be risk factors for violence perpetrated by patients or family members. Limited professional experience not underpinned by appropriate communication skills, combined with inability to anticipate patient needs (Bottega & Palese, 2020), do not enable to notice the initial signs of aggression and consequently prevent it. Other studies have shown that specific interventions aimed at raising nurses' awareness about risk factors, such as young age and limited experience, are essential in reducing aggressive behaviors in patients and their families (Hill et al., 2015; Shi et al., 2017).

Organizational and environmental factors are the most frequently reported risk factors of violence perpetrated by patients. In particular, the emergency department is the setting where WPV is reported to occur by most studies. Understaffing and high workloads are reported as the most frequent risk factors for WPV. Staff shortages that have persisted for decades in hospitals have dramatically worsened over the past 2 years due to the COVID‐19 pandemic. Patient‐to‐nurse ratios vary widely in hospitals, and when nurses have to care for an excessively high number of patients, the chances of causing harm to patients are high (Khera et al., 2021; Lasater et al., 2021). For this reason, the phenomenon of assaults perpetrated by patients may have increased in this period due to the critical shortage of nurses and the increased workload.

Long waiting times in the emergency department (Morphet et al., 2014) associated with patients' unrealistic expectations has also been described as a major risk factor of physical and verbal aggression. In these cases, waiting time management strategies providing timely information and assistance to users, and specific education programmes for emergency personnel, could reduce the cases of aggression (Gillespie et al., 2014; Touzet et al., 2019). The lack of protocols and policies for the management and prevention of violence, the absence of dedicated communication channels and specific means to inform managers and administrators about episodes of violence are described by several studies (Babiarczyk et al., 2019; Jenkins et al., 1998). These shortcomings often occur in contexts where the incidence of violence against nurses is high (Cannavo et al., 2019). In addition, characteristics of the perpetrators, such as their mental status, clinical conditions and alcohol or drug abuse, have been identified as common risk factors of WPV. Greater awareness of the role played by these characteristics in WPV and advanced skills that enable to adequately approach these types of patients could help to predict, prevent, or limit the development of aggressive behaviors (Liu et al., 2019).

Nursing students also suffer violence perpetrated by patients and their families. Likewise, personal characteristics (e.g., gender, age and marital status) and organizational factors (e.g., attending emergency department internship) have been identified as risk factors. Teachers and clinical preceptors have a great responsibility in ensuring a safe learning environment. When personal characteristics and organizational and environmental factors are recognized as risk factors, they must be considered, together with the inherent vulnerability of learners, so that actions that protect students during their clinical learning programme are in place (Seibel & Fehr, 2018; Tee et al., 2016).

The consequences of WPV impact specifically on individual nurses, and generally on the health organization. These affect the quality of care provided, professional life and the emotional, psychophysical and physical well‐being of nurses and nursing students. Physical and verbal assaults are related to burnout in each of its three dimensions (Laschinger et al., 2010; Wu et al., 2020; Yang et al., 2018). In this regard, the availability of follow‐up programmes for WPV victims, counselling and discussion with hospital administrators have been found to reduce emotional exhaustion and depersonalization, and increase personal accomplishment (Vincent‐Höper et al., 2020). In addition, burnout generated by violence reduces nurses' level of attention when providing care (Al‐Ghabeesh & Qattom, 2019a), increasing the likelihood of errors and putting patients' safety and health at risk. On the other side, the poor quality of the care is perceived negatively by patients, who may not feel actively involved and receive unsatisfactory responses to their needs due to distracted nursing care.

### 5.3 Economic consequences of workplace violence

Very few studies examined the economic consequences of violence but showed how costs incurred by health institutions rise significantly due to compensation measures for professionals who become victims of violence, their reintegration into the workplace and increased turnover. As in other studies (Jeong & Kim, 2018; Olsen et al., 2017), workplace violence is a significant cause of turnover intent. Constant turnover is an impediment to effective teamwork and cohesion among colleagues, or even worse, it may reinforce any negative attitudes that may harbour in senior staff (Van Bogaert et al., 2017). Furthermore, some consequences of violence, such as burnout, depersonalization and physical harm, also increase intention of turnover and intention to leave the profession that can lead to enormous costs for the health care organizations that have to cope with this phenomenon.

### 5.4 Preventing and managing workplace violence

Nurse leaders are in the position to promote a culture of safety that prioritizes the health, safety and wellbeing of their staff, patients and visitors. Health managers should promote policies that refuse violence as an inevitable part of professional practice and allocate resources for the prevention and management of violence and bullying (Johnson et al., 2018; Pariona‐Cabrera et al., 2020). Some studies identified strategies to manage and prevent WPV episodes at different levels. For instance, allocating considerable funds to the prevention and management of WPV (Morphet et al., 2019), increasing staff numbers to prevent and manage WPV (Morphet et al., 2018), developing guidance materials evidence‐based, focusing on education and training of staff to manage WPV (Geoffrion et al., 2020), implementing monitoring, responding and reporting systems (Burkoski et al., 2019; Ramacciati et al., 2021), sharing information between health services and other agencies and improving communication abilities (Collins, 2021) and implementing an effective security staff (Morphet et al., 2019).

## 6 CONCLUSIONS

The results of this review bring to light critical issues often left unaddressed, especially where episodes of violence are very frequent. WPV prevention and management programmes and proactive commitment are essential to reduce WPV and its consequences. Nursing leaders must explore and implement practices towards mitigating violence against nurses. Action research is needed to engage in a cycle of continuous improvement that supports eliminating violence in the health care sector.

Initiatives for the health and safety of nurses that establish objectives and responsibilities to monitor and curb WPV, and reports describing the outcomes of the measures adopted to prevent and manage episodes of violence should be on the agenda of every health administration. There is sufficient evidence for nurse managers to ensure that nurses and all health care professionals feel protected and safeguarded from verbal or physical abuse, and work in environments that ensure maximum safety for everyone.

### 6.1 Limitations

This review included papers about WPV suffered by nurses and nursing students excluding other health professions. Despite the inclusion criteria for this study being wide, limitations can be found in language restrictions (English and Italian) that may have excluded significant studies written in other languages. Most of the studies included in this review were from the North American Continent and Europe, which limits the generalizability of our conclusions.

### 6.2 Implications for nursing management

The predictors and consequences of WPV identified through this review constitute the body of knowledge necessary for nurse managers to develop and implement actions to manage or prevent WPV effectively.

Therefore, there is sufficient evidence for nurse managers to contribute to the development of a positive safety culture and awareness, putting at its centre the health, safety and wellbeing of health personnel, patients and visitors. Nurse managers must promote policies that decline violence as an inevitable part of nursing practice and invest resources to neutralize the onset of episodes of violence and transform it into an opportunity for professional and cultural development.

Evidence‐based management of violence can contribute to implementing actions that ensure a violence‐free working environment through permanent monitoring and reporting systems.

Furthermore, this message on the impact of WPV in health care must also be spread to a broader audience to promote and support change effectively.

## CONFLICT OF INTEREST

The authors of this manuscript have no competing interests as defined by the editorial policy of *Journal of Nursing Management*. They moreover have no other interests that may have influenced the results and discussion of this paper.

## ETHICS STATEMENT

Since this is a review of the literature, no ethics approval is required.

## AUTHORS' CONTRIBUTIONS

Nicola Pagnucci: Conceptualization, Writing‐Original draft preparation.

Giulia Ottonello: Analysis, Writing‐Original draft preparation.

Davide Capponi: Analysis, visualization.

Gianluca Catania: Supervision of the review process.

Milko Zanini: Supervision of the analysis.

Giuseppe Aleo: Reviewing and editing final draft.

Fiona Timmins: Reviewing and editing final draft.

Loredana Sasso: Overall supervision.

Annamaria Bagnasco: Conceptualization and overall supervision.

## Data Availability

Authors do not wish to share the data.

## References

[jonm13635-bib-0001] Abou‐ElWafa, H. S. , El‐Gilany, A.‐H. , Abd‐El‐Raouf, S. E. , Abd‐Elmouty, S. M. , & El‐Sayed Hassan El‐Sayed, R. (2014). Workplace violence against emergency versus non‐emergency nurses in Mansoura University Hospitals, Egypt. Journal of Interpersonal Violence, 30(5), 857–872. 10.1177/0886260514536278 24970863

[jonm13635-bib-0002] AbuAlRub, R. F. , & Al Khawaldeh, A. T. (2014). Workplace physical violence among hospital nurses and physicians in underserved areas in Jordan. Journal of Clinical Nursing, 23(13–14), 1937–1947. 10.1111/jocn.12473 24354354

[jonm13635-bib-0003] AbuAlRub, R. F. , & Al‐Asmar, A. H. (2011). Physical violence in the workplace among Jordanian hospital nurses. Journal of Transcultural Nursing, 22(2), 157–165. 10.1177/1043659610395769 21311085

[jonm13635-bib-0004] AbuAlRub, R. F. , Khalifa, M. F. , & Habbib, M. B. (2007). Workplace violence among Iraqi hospital nurses. Journal of Nursing Scholarship, 39(3), 281–288. 10.1111/j.1547-5069.2007.00181.x 17760803

[jonm13635-bib-0005] Aksakal, F. N. , Karasahin, E. F. , Dikmen, A. U. , Avci, E. , & Ozkan, S. (2015). Workplace physical violence, verbal violence, and mobbing experienced by nurses at a university hospital. Turkish Journal of Medical Sciences, 45(6), 1360–1368. 10.3906/sag-1405-65 26775395

[jonm13635-bib-0006] Alameddine, M. , Mourad, Y. , & Dimassi, H. (2015). A National Study on Nurses' exposure to occupational violence in Lebanon: Prevalence, consequences and associated factors. PLoS ONE, 10(9), e0137105. 10.1371/journal.pone.0137105 26355686 PMC4565636

[jonm13635-bib-0007] Al‐Ghabeesh, S. H. , & Qattom, H. (2019a). RETRACTED ARTICLE: Workplace bullying and its preventive measures and productivity among emergency department nurses. Israel Journal Health Policy Research, 8(1), 44. 10.1186/s13584-019-0314-8 PMC6524255 31101071

[jonm13635-bib-0008] Al‐Ghabeesh, S. H. , & Qattom, H. (2019b). Workplace bullying and its preventive measures and productivity among emergency department nurses. BMC Health Services Research, 19(1), 445. 10.1186/s12913-019-4268-x 31269990 PMC6607587

[jonm13635-bib-0009] Al‐Omari, H. (2015). Physical and verbal workplace violence against nurses in Jordan. International Nursing Review, 62(1), 111–118. 10.1111/inr.12170 25626758

[jonm13635-bib-0010] Anusiewicz, C. V. , Ivankova, N. V. , Swiger, P. A. , Gillespie, G. L. , Li, P. , & Patrician, P. A. (2020). How does workplace bullying influence nurses' abilities to provide patient care? A nurse perspective. Journal of Clinical Nursing, 29(21–22), 4148–4160. 10.1111/jocn.15443 32757394 PMC8040339

[jonm13635-bib-0011] Aromataris, E. , & Munn, Z. (2020). JBI manual for evidence synthesis. 10.46658/jbimes-20-01

[jonm13635-bib-0012] Avander, K. , Heikki, A. , Bjerså, K. , & Engström, M. (2016). Trauma Nurses' experience of workplace violence and threats. Journal of Trauma Nursing, 23(2), 51–57. 10.1097/jtn.0000000000000186 26953531

[jonm13635-bib-0013] Babiarczyk, B. , Turbiarz, A. , Tomagová, M. , Zeleníková, R. , Önler, E. , & Sancho Cantus, D. (2019). Violence against nurses working in the health sector in five European countries—Pilot study. International Journal of Nursing Practice, 25(4), e12744. 10.1111/ijn.12744 31172630

[jonm13635-bib-0014] Baby, M. , Glue, P. , & Carlyle, D. (2014). ‘Violence is not part of our job’: A thematic analysis of psychiatric mental health Nurses' experiences of patient assaults from a New Zealand perspective. Issues in Mental Health Nursing, 35(9), 647–655. 10.3109/01612840.2014.892552 25162186

[jonm13635-bib-0015] Bambi, S. , Becattini, G. , Giusti, G. D. , Mezzetti, A. , Guazzini, A. , & Lumini, E. (2014). Lateral hostilities among nurses employed in intensive care units, emergency departments, operating rooms, and emergency medical services. Dimensions of Critical Care Nursing, 33(6), 347–354. 10.1097/dcc.0000000000000077 25280203

[jonm13635-bib-0016] Bambi, S. , Guazzini, A. , Piredda, M. , Lucchini, A. , De Marinis, M. G. , & Rasero, L. (2019). Negative interactions among nurses: An explorative study on lateral violence and bullying in nursing work settings. Journal of Nursing Management, 27(4), 749–757. 10.1111/jonm.12738 30506602

[jonm13635-bib-0017] Bardakçı, E. , & Günüşen, N. P. (2014). Influence of workplace bullying on Turkish Nurses' psychological distress and nurses' reactions to bullying. Journal of Transcultural Nursing, 27(2), 166–171. 10.1177/1043659614549073 25193340

[jonm13635-bib-0018] Basfr, W. , Hamdan, A. , & Al‐Habib, S. (2019). Workplace violence against nurses in psychiatric hospital settings: Perspectives from Saudi Arabia. Sultan Qaboos University Medical Journal [SQUMJ], 19(1), 19–e25. 10.18295/squmj.2019.19.01.005 PMC6544070 31198591

[jonm13635-bib-0019] Beattie, J. , Innes, K. , Griffiths, D. , & Morphet, J. (2020). Workplace violence: Examination of the tensions between duty of care, worker safety, and zero tolerance. Health Care Management Review, 45(3), E13–E22. 10.1097/hmr.0000000000000286 32358237

[jonm13635-bib-0020] Benveniste, K. A. , Hibbert, P. D. , & Runciman, W. B. (2005). Violence in health care: The contribution of the Australian patient Safety Foundation to incident monitoring and analysis. Medical Journal of Australia, 183(7), 348–351. 10.5694/j.1326-5377.2005.tb07081.x 16201951

[jonm13635-bib-0021] Berlanda, S. , Pedrazza, M. , Fraizzoli, M. , & de Cordova, F. (2019). Addressing risks of violence against healthcare staff in emergency departments: The effects of job satisfaction and attachment style. BioMed Research International, 2019, 1–12. 10.1155/2019/5430870 PMC6558649 31275976

[jonm13635-bib-0022] Bimenyimana, E. , Poggenpoel, M. , Myburgh, C. , & Van Niekerk, V. (2009). The lived experience by psychiatric nurses of aggression and violence from patients in a Gauteng psychiatric institution. Curationis, 32(3). 10.4102/curationis.v32i3.1218 20225739

[jonm13635-bib-0023] Blackstock, S. , Harlos, K. , Macleod, M. L. P. , & Hardy, C. L. (2015). The impact of organisational factors on horizontal bullying and turnover intentions in the nursing workplace. Journal of Nursing Management, 23(8), 1106–1114. 10.1111/jonm.12260 25370741

[jonm13635-bib-0024] Bloom, E. M. (2019). Horizontal violence among nurses: Experiences, responses, and job performance. Nursing Forum, 54(1), 77–83. 10.1111/nuf.12300 30332520

[jonm13635-bib-0025] Boafo, I. M. , & Hancock, P. (2017). Workplace Violence Against Nurses. SAGE Open, 7(1). 10.1177/2158244017701187 PMC5047339 27708820

[jonm13635-bib-0026] Bottega, M. , & Palese, A. (2020). Anticipated nursing care: Findings from a qualitative study. BMC Nursing, 19(1), 93. 10.1186/s12912-020-00486-y 33041658 PMC7541304

[jonm13635-bib-0027] Brooks Carthon, J. M. , Hatfield, L. , Brom, H. , Houton, M. , Kelly‐Hellyer, E. , Schlak, A. , & Aiken, L. H. (2021). System‐level improvements in work environments Lead to lower nurse burnout and higher patient satisfaction. Journal of Nursing Care Quality, 36(1), 7–13. 10.1097/NCQ.0000000000000475 32102025 PMC7483185

[jonm13635-bib-0144] Budin, W. C. , Brewer, C. S. , Chao, Y.‐Y. , & Kovner, C. (2013). Verbal Abuse From Nurse Colleagues and Work Environment of Early Career Registered Nurses. Journal of Nursing Scholarship, 45(3), 308–316. 10.1111/jnu.12033 23627991

[jonm13635-bib-0028] Burkoski, V. , Farshait, N. , Yoon, J. , Clancy, P. V. , Fernandes, K. , Howell, M. R. , Solomon, S. , Orrico, M. E. , & Collins, B. E. (2019). Violence prevention: Technology‐enabled therapeutic intervention. Nursing Leadership (Toronto, Ont.), 32(SP, 58–70. 10.12927/cjnl.2019.25814 31099747

[jonm13635-bib-0029] Çam, H. H. , & Ustuner Top, F. (2021). Workplace violence against nurses working in the public hospitals in Giresun, Turkey: Prevalence, risk factors, and quality of life consequences. In Perspectives in psychiatric care. 10.1111/ppc.12978 34860413

[jonm13635-bib-0030] Cannavo, M. , La Torre, F. , Sestili, C. , La Torre, G. , & Fioravanti, M. (2019). Work related violence as a predictor of stress and correlated disorders in emergency department healthcare professionals. La Clinica Terapeutica, 170(2), e110–e123. 10.7417/CT.2019.2120 30993307

[jonm13635-bib-0031] Ceballos, J. B. , Frota, O. P. , Nunes, H. F. S. S. , Ávalos, P. L. , Krügel, C. d. C. , Ferreira Júnior, M. A. , & Teston, E. F. (2020). Physical violence and verbal abuse against nurses working with risk stratification: Characteristics, related factors, and consequences. Revista Brasileira de Enfermagem, 73(suppl 5), e20190882. 10.1590/0034-7167-2019-0882 33338160

[jonm13635-bib-0032] Çelik, Y. , & Çelik, S. Ş. (2007). Sexual harassment against nurses in Turkey. Journal of Nursing Scholarship, 39(2), 200–206. 10.1111/j.1547-5069.2007.00168.x 17535323

[jonm13635-bib-0033] Chapman, R. , & Styles, I. (2006). An epidemic of abuse and violence: Nurse on the front line. Accident and Emergency Nursing, 14(4), 245–249. 10.1016/j.aaen.2006.08.004 17064902

[jonm13635-bib-0034] Chatziioannidis, I. , Bascialla, F. G. , Chatzivalsama, P. , Vouzas, F. , & Mitsiakos, G. (2018). Prevalence, causes and mental health impact of workplace bullying in the neonatal intensive care unit environment. BMJ Open, 8(2), e018766. 10.1136/bmjopen-2017-018766 PMC5855440 29478015

[jonm13635-bib-0035] Cheung, T. , & Yip, P. S. F. (2017). Workplace violence towards nurses in Hong Kong: Prevalence and correlates. BMC Public Health, 17(1), 196. 10.1186/s12889-017-4112-3 28196499 PMC5310001

[jonm13635-bib-0139] Christopher, L. R. L. M. S. (1998). Staff assaults and injuries in a psychiatric hospital as a function of three attitudinal variables. Issues in Mental Health Nursing, 19(3), 277–289. 10.1080/016128498249079 9661378

[jonm13635-bib-0036] Clarke, C. M. , Kane, D. J. , Rajacich, D. L. , & Lafreniere, K. D. (2012). Bullying in undergraduate clinical nursing education. The Journal of Nursing Education, 51(5), 269–276. 10.3928/01484834-20120409-01 22495922

[jonm13635-bib-0037] Collins, R. (2021). Protect the nurse, protect the practice: Effective communication is the foundation for keeping nurses safe. Healthcare Management Forum, 34(4), 200–204. 10.1177/08404704211022144 34128425

[jonm13635-bib-0038] Coskun Cenk, S. (2019). An analysis of the exposure to violence and burnout levels of ambulance staff. Turkish Journal of Emergency Medicine, 19(1), 21–25. 10.1016/j.tjem.2018.09.002 30793061 PMC6370911

[jonm13635-bib-0039] Dangal, G. , Bhandari, T. R. , & Pandey, M. (2018). Workplace violence and its associated factors among nurses. Journal of Nepal Health Research Council, 15(3), 235–241. 10.3126/jnhrc.v15i3.18847 29353895

[jonm13635-bib-0140] Difazio, R. L. , Vessey, J. A. , Buchko, O. A. , Chetverikov, D. V. , Sarkisova, V. A. , & Serebrennikova, N. V. (2019). The incidence and outcomes of nurse bullying in the Russian Federation. International Nursing Review, 66(1), 94–103. 10.1111/inr.12479 30192382

[jonm13635-bib-0040] Edmonson, C. , & Zelonka, C. (2019). Our own worst enemies. Nursing Administration Quarterly, 43(3), 274–279. 10.1097/naq.0000000000000353 31162347 PMC6716575

[jonm13635-bib-0041] Estryn‐Behar, M. , van der Heijden, B. , Camerino, D. , Fry, C. , Le Nezet, O. , Conway, P. M. , & Hasselhorn, H. M. (2008). Violence risks in nursing—Results from the European 'NEXT' Study. Occupational Medicine, 58(2), 107–114. 10.1093/occmed/kqm142 18211910

[jonm13635-bib-0042] Evers, W. , Tomic, W. , & Brouwers, A. (2002). Aggressive behaviour and burnout among staff of homes for the elderly. International Journal of Mental Health Nursing, 11(1), 2–9. 10.1046/j.1440-0979.2002.00219.x 12400101

[jonm13635-bib-0043] Fafliora, E. , Bampalis, V. G. , Zarlas, G. , Sturaitis, P. , Lianas, D. , & Mantzouranis, G. (2016). Workplace violence against nurses in three different Greek healthcare settings. Work, 53(3), 551–560. 10.3233/wor-152225 26835853

[jonm13635-bib-0044] Farrell, G. A. , Shafiei, T. , & Chan, S.‐P. (2014). Patient and visitor assault on nurses and midwives: An exploratory study of employer ‘protective’ factors. International Journal of Mental Health Nursing, 23(1), 88–96. 10.1111/inm.12002 23279321

[jonm13635-bib-0045] Favaro, A. , Wong, C. , & Oudshoorn, A. (2021). Relationships among sex, empowerment, workplace bullying and job turnover intention of new graduate nurses. Journal of Clinical Nursing, 30(9–10), 1273–1284. 10.1111/jocn.15671 33476435

[jonm13635-bib-0046] Ferri, P. , Silvestri, M. , Artoni, C. , & Di Lorenzo, R. (2016). Workplace violence in different settings and among various health professionals in an Italian general hospital: A cross‐sectional study. Psychology Research and Behavior Management, 9, 263, S114870–275. 10.2147/prbm 27729818 PMC5042196

[jonm13635-bib-0047] Fontes, K. B. , Alarcão, A. C. J. , Santana, R. G. , Pelloso, S. M. , & Barros Carvalho, M. D. (2018). Relationship between leadership, bullying in the workplace and turnover intention among nurses. Journal of Nursing Management, 27(3), 535–542. 10.1111/jonm.12708 30136314

[jonm13635-bib-0048] Franz, S. , Zeh, A. , Schablon, A. , Kuhnert, S. , & Nienhaus, A. (2010). Aggression and violence against health care workers in Germany—A cross sectional retrospective survey. BMC Health Services Research, 10(1). 10.1186/1472-6963-10-51 PMC2837654 20184718

[jonm13635-bib-0049] Fujimoto, H. , Hirota, M. , Kodama, T. , Greiner, C. , & Hashimoto, T. (2017). Violence exposure and resulting psychological effects suffered by psychiatric visiting nurses in Japan. Journal of Psychiatric and Mental Health Nursing, 24(8), 638–647. 10.1111/jpm.12412 28840659

[jonm13635-bib-0050] Gacki‐Smith, J. , Juarez, A. M. , Boyett, L. , Homeyer, C. , Robinson, L. , & MacLean, S. L. (2009). Violence against nurses working in US emergency departments. JONA: The Journal of Nursing Administration, 39(7/8), 340–349. 10.1097/NNA.0b013e3181ae97db 19641432

[jonm13635-bib-0051] Galián Muñoz, I. , Llor‐Esteban, B. , & Ruiz Hernández, J. A. (2014). Violence against nursing staff in hospital emergency services: Risk factors and consequences. Emergencias, 26(3), 163–170.

[jonm13635-bib-0052] Galian‐Munoz, I. , Llor‐Esteban, B. , & Ruiz‐Hernández, J. A. (2014). Violence against nursing staff in hospital emergency services: Risk factors and consequences. Emergencias, 26, 163–170.

[jonm13635-bib-0053] Geoffrion, S. , Hills, D. J. , Ross, H. M. , Pich, J. , Hill, A. T. , Dalsbo, T. K. , Riahi, S. , Martinez‐Jarreta, B. , & Guay, S. (2020). Education and training for preventing and minimizing workplace aggression directed toward healthcare workers. Cochrane Database of Systematic Reviews, 9, CD011860. 10.1002/14651858.CD011860.pub2 32898304 PMC8094156

[jonm13635-bib-0054] Gerberich, S. G. (2004). An epidemiological study of the magnitude and consequences of work related violence: The Minnesota Nurses' study. Occupational and Environmental Medicine, 61(6), 495–503. 10.1136/oem.2003.007294 15150388 PMC1763639

[jonm13635-bib-0055] Gerberich, S. G. , Church, T. R. , McGovern, P. M. , Hansen, H. , Nachreiner, N. M. , Geisser, M. S. , Ryan, A. D. , Mongin, S. J. , Watt, G. D. , & Jurek, A. (2005). Risk factors for work‐related assaults on nurses. Epidemiology, 16(5), 704–709. 10.1097/01.ede.0000164556.14509.a3 16135952

[jonm13635-bib-0056] Gillespie, G. L. , Gates, D. M. , Kowalenko, T. , Bresler, S. , & Succop, P. (2014). Implementation of a comprehensive intervention to reduce physical assaults and threats in the emergency department. Journal of Emergency Nursing, 40(6), 586–591. 10.1016/j.jen.2014.01.003 24612728

[jonm13635-bib-0057] Goedhart, N. S. , van Oostveen, C. J. , & Vermeulen, H. (2017). The effect of structural empowerment of nurses on quality outcomes in hospitals: A scoping review. Journal of Nursing Management, 25(3), 194–206. 10.1111/jonm.12455 28078745

[jonm13635-bib-0058] Grainger, C. , & Whiteford, H. (1993). Assault on staff in psychiatric hospitals: A safety issue. Australian and New Zealand Journal of Psychiatry, 27(2), 324–328. 10.3109/00048679309075785 8363544

[jonm13635-bib-0059] Hahn, S. , Müller, M. , Needham, I. , Dassen, T. , Kok, G. , & Halfens, R. J. G. (2010). Factors associated with patient and visitor violence experienced by nurses in general hospitals in Switzerland: A cross‐sectional survey. Journal of Clinical Nursing, 19(23–24), 3535–3546. 10.1111/j.1365-2702.2010.03361.x 20958803

[jonm13635-bib-0060] Hamdan, M. , & Hamra, A. a. A. (2017). Burnout among workers in emergency Departments in Palestinian hospitals: Prevalence and associated factors. BMC Health Services Research, 17(1), 407. 10.1186/s12913-017-2356-3 28619081 PMC5472878

[jonm13635-bib-0061] Hampton, D. , & Rayens, M. K. (2019). Impact of psychological empowerment on workplace bullying and intent to leave. JONA: The Journal of Nursing Administration, 49(4), 179–185. 10.1097/nna.0000000000000735 30829723

[jonm13635-bib-0062] Hanohano, C. J. O. (2017). Physical assault, perceived stress, coping, and attitudes toward assault experienced by psychiatric nurses and their intent to leave Azusa Pacific University. Azusa, California.

[jonm13635-bib-0063] Hartin, P. , Birks, M. , & Lindsay, D. (2020). Bullying in nursing: How has it changed over 4 decades? Journal of Nursing Management, 28(7), 1619–1626. 10.1111/jonm.13117 32745349

[jonm13635-bib-0064] Hassankhani, H. , & Soheili, A. (2017). Zero‐tolerance policy: The last way to curb workplace violence against nurses in Iranian healthcare system. Journal of Caring Sciences, 6(1), 1–3. 10.15171/jcs.2017.001 28299292 PMC5348658

[jonm13635-bib-0138] Havaei, F. , Astivia, O. L. O. , & MacPhee, M. (2020). The impact of workplace violence on medical‐surgical nurses’ health outcome: A moderated mediation model of work environment conditions and burnout using secondary data. International Journal of Nursing Studies, 109, 10.1016/j.ijnurstu.2020.103666 32592889

[jonm13635-bib-0065] Heslop, L. , Kim, Y. , Lee, E. , & Lee, H. (2019). Association between workplace bullying and burnout, professional quality of life, and turnover intention among clinical nurses. PLOS One, 14(12), e0226506. 10.1371/journal.pone.0226506 31860673 PMC6924657

[jonm13635-bib-0066] Higgins, B. L. , & MacIntosh, J. (2010). Operating room nurses' perceptions of the effects of physician‐perpetrated abuse. International Nursing Review, 57(3), 321–327. 10.1111/j.1466-7657.2009.00767.x 20796061

[jonm13635-bib-0067] Hill, A. K. , Lind, M. A. , Tucker, D. , Nelly, P. , & Daraiseh, N. (2015). Measurable results: Reducing staff injuries on a specialty psychiatric unit for patients with developmental disabilities. Work, 51(1), 99–111. 10.3233/WOR-152014 25835723

[jonm13635-bib-0068] Hong, S. , Kim, H. , Nam, S. , Wong, J. Y. H. , & Lee, K. (2021). Nurses' post‐traumatic stress symptoms and growth by perceived workplace bullying: An online cross‐sectional study. Journal of Nursing Management, 29(5), 1338–1347. 10.1111/jonm.13275 33486839

[jonm13635-bib-0069] Hutton, S. , & Gates, D. (2008). Workplace incivility and productivity losses among direct care staff. AAOHN Journal, 56(4), 168–175. 10.3928/08910162-20080401-01 18444405

[jonm13635-bib-0070] ILO‐International Labour Organization . (2003). Code of practice on workplace violence in services sectors and measures to combat this phenomenon. Geneva

[jonm13635-bib-0071] Jafree, S. R. (2017). Workplace violence against women nurses working in two public sector hospitals of Lahore, Pakistan. Nursing Outlook, 65(4), 420–427. 10.1016/j.outlook.2017.01.008 28343713

[jonm13635-bib-0072] Jakobsson, J. , Örmon, K. , Berthelsen, H. , & Axelsson, M. (2021). Workplace violence from the perspective of hospital ward managers in Sweden: A qualitative study. Journal of Nursing Management. 10.1111/jonm.13423 34273122

[jonm13635-bib-0073] Jaradat, Y. , Nielsen, M. B. , Kristensen, P. , Nijem, K. , Bjertness, E. , Stigum, H. , & Bast‐Pettersen, R. (2016). Workplace aggression, psychological distress, and job satisfaction among Palestinian nurses: A cross‐sectional study. Applied Nursing Research, 32, 190–198. 10.1016/j.apnr.2016.07.014 27969027

[jonm13635-bib-0074] Jenkins, M. G. , Rocke, L. G. , McNicholl, B. P. , & Hughes, D. M. (1998). Violence and verbal abuse against staff in accident and emergency departments: A survey of consultants in the UK and the Republic of Ireland. Emergency Medicine Journal, 15(4), 262–265. 10.1136/emj.15.4.262 PMC1343141 9681312

[jonm13635-bib-0075] Jeong, I. Y. , & Kim, J. S. (2018). The relationship between intention to leave the hospital and coping methods of emergency nurses after workplace violence. Journal of Clinical Nursing, 27(7–8), 1692–1701. 10.1111/jocn.14228 29266478

[jonm13635-bib-0076] Johnson, A. , Nguyen, H. , Groth, M. , & White, L. (2018). Workplace aggression and organisational effectiveness: The mediating role of employee engagement. Australian Journal of Management, 43(4), 614–631. 10.1177/0312896218768378

[jonm13635-bib-0077] Khera, R. , Liu, Y. , de Lemos, J. A. , Das, S. R. , Pandey, A. , Omar, W. , Kumbhani, D. J. , Girotra, S. , Yeh, R. W. , Rutan, C. , Walchok, J. , Lin, Z. , Bradley, S. M. , Velazquez, E. J. , Churchwell, K. B. , Nallamothu, B. K. , Krumholz, H. M. , & Curtis, J. P. (2021). Association of COVID‐19 hospitalization volume and case growth at US hospitals with patient outcomes. The American Journal of Medicine, 134(11), 1380, e1383–1388. 10.1016/j.amjmed.2021.06.034 34343515 PMC8325555

[jonm13635-bib-0078] Kobayashi, Y. , Oe, M. , Ishida, T. , Matsuoka, M. , Chiba, H. , & Uchimura, N. (2020). Workplace violence and its effects on burnout and secondary traumatic stress among mental healthcare nurses in Japan. International Journal of Environmental Research and Public Health, 17(8). 10.3390/ijerph17082747 PMC7215457 32316142

[jonm13635-bib-0079] Kowalenko, T. , Gates, D. , Gillespie, G. L. , Succop, P. , & Mentzel, T. K. (2013). Prospective study of violence against ED workers. The American Journal of Emergency Medicine, 31(1), 197–205. 10.1016/j.ajem.2012.07.010 23000325

[jonm13635-bib-0080] Kozakova, R. , Bužgová, R. , & Zeleníková, R. (2018). Mobbing of nurses: Prevalence, forms and psychological consequences in the moravian‐silesian region. Ceskoslovenska Psychologie, 62(4), 316–329.

[jonm13635-bib-0081] Lasater, K. B. , Aiken, L. H. , Sloane, D. M. , French, R. , Martin, B. , Reneau, K. , Alexander, M. , & McHugh, M. D. (2021). Chronic hospital nurse understaffing meets COVID‐19: An observational study. BMJ Quality and Safety, 30(8), 639–647. 10.1136/bmjqs-2020-011512 PMC7443196 32817399

[jonm13635-bib-0082] Laschinger, H. K. S. , & Grau, A. L. (2012). The influence of personal dispositional factors and organizational resources on workplace violence, burnout, and health outcomes in new graduate nurses: A cross‐sectional study. International Journal of Nursing Studies, 49(3), 282–291. 10.1016/j.ijnurstu.2011.09.004 21978860

[jonm13635-bib-0083] Laschinger, H. K. S. , Grau, A. L. , Finegan, J. , & Wilk, P. (2010). New graduate nurses experiences of bullying and burnout in hospital settings. Journal of Advanced Nursing, 66(12), 2732–2742. 10.1111/j.1365-2648.2010.05420.x 20722806

[jonm13635-bib-0084] Lash, A. A. , Kulakac, O. , Buldukoglu, K. , & Kukulu, K. (2006). Verbal abuse of nursing and midwifery students in clinical settings in Turkey. The Journal of Nursing Education, 45(10), 396–403. 10.3928/01484834-20061001-04 17058694

[jonm13635-bib-0085] Levin, P. F. , Hewitt, J. B. , & Misner, S. T. (1998). Insights of nurses about assault in hospital‐based emergency departments. Image: The Journal of Nursing Scholarship, 30(3), 249–254. 10.1111/j.1547-5069.1998.tb01300.x 9753840

[jonm13635-bib-0086] Liu, J. , Gan, Y. , Jiang, H. , Li, L. , Dwyer, R. , Lu, K. , Yan, S. , Sampson, O. , Xu, H. , Wang, C. , Zhu, Y. , Chang, Y. , Yang, Y. , Yang, T. , Chen, Y. , Song, F. , & Lu, Z. (2019). Prevalence of workplace violence against healthcare workers: A systematic review and meta‐analysis. Occupational and Environmental Medicine, 76(12), 927–937. 10.1136/oemed-2019-105849 31611310

[jonm13635-bib-0142] McKenna, B. G. , Smith, N. A. , Poole, S. J. , & Coverdale, J. H. (2003). Horizontal violence: experiences of Registered Nurses in their first year of practice. Journal of Advanced Nursing, 42(1), 90–96. 10.1046/j.1365-2648.2003.02583.x 12641816

[jonm13635-bib-0087] Merecz, D. , Rymaszewska, J. , Mościcka, A. , Kiejna, A. , & Jarosz‐Nowak, J. (2020). Violence at the workplace—A questionnaire survey of nurses. European Psychiatry, 21(7), 442–450. 10.1016/j.eurpsy.2006.01.001 16530389

[jonm13635-bib-0088] Morphet, J. , Griffiths, D. , Plummer, V. , Innes, K. , Fairhall, R. , & Beattie, J. (2014). At the crossroads of violence and aggression in the emergency department: Perspectives of Australian emergency nurses. Australian Health Review, 38(2), 194–201. 10.1071/ah13189 24670224

[jonm13635-bib-0089] Morphet, J. , Griffiths, D. , Beattie, J. , Velasquez Reyes, D. , & Innes, K. (2018). Prevention and management of occupational violence and aggression in healthcare: A scoping review. Collegian, 25(6), 621–632. 10.1016/j.colegn.2018.04.003

[jonm13635-bib-0090] Morphet, J. , Griffiths, D. , Beattie, J. , & Innes, K. (2019). Managers experiences of prevention and management of workplace violence against health care staff: A descriptive exploratory study. Journal of Nursing Management, 27(4), 781–791. 10.1111/jonm.12761 30784135

[jonm13635-bib-0091] Moura, L. N. , Camponogara, S. , Santos, J. L. G. D. , Gasparino, R. C. , Silva, R. M. D. , & Freitas, E. D. O. (2020). Structural empowerment of nurses in the hospital setting. Revista Latino‐Americana de Enfermagem, 28, e3373. 10.1590/1518-8345.3915.3373 33174992 PMC7647415

[jonm13635-bib-0092] Najafi, F. , Fallahi‐Khoshknab, M. , Ahmadi, F. , Dalvandi, A. , & Rahgozar, M. (2018). Antecedents and consequences of workplace violence against nurses: A qualitative study. Journal of Clinical Nursing, 27(1–2), e116–e128. 10.1111/jocn.13884 28514533

[jonm13635-bib-0093] Nguluwe, B. C. J. , Havenga, Y. , & Sengane, M. L. M. (2016). Violence experienced by nurses working in acute care psychiatric wards at a Gauteng hospital. Africa Journal of Nursing and Midwifery, 16(1), 60–74. 10.25159/2520-5293/1488

[jonm13635-bib-0094] Ogundipe, K. O. , Etonyeaku, A. C. , Adigun, I. , Ojo, E. O. , Aladesanmi, T. , Taiwo, J. O. , & Obimakinde, O. S. (2013). Violence in the emergency department: A multicentre survey of nurses perceptions in Nigeria. Emergency Medicine Journal, 30(9), 758–762. 10.1136/emermed-2012-201541 23038694

[jonm13635-bib-0095] Olsen, E. , Bjaalid, G. , & Mikkelsen, A. (2017). Work climate and the mediating role of workplace bullying related to job performance, job satisfaction, and work ability: A study among hospital nurses. Journal of Advanced Nursing, 73(11), 2709–2719. 10.1111/jan.13337 28512986

[jonm13635-bib-0096] Pai, H.‐C. , & Lee, S. (2011). Risk factors for workplace violence in clinical registered nurses in Taiwan. Journal of Clinical Nursing, 20(9–10), 1405–1412. 10.1111/j.1365-2702.2010.03650.x 21492284

[jonm13635-bib-0097] Palese, A. , Spelten, E. , Thomas, B. , OMeara, P. , van Vuuren, J. , & McGillion, A. (2020). Violence against emergency department nurses; can we identify the perpetrators? PLoS ONE, 15(4). 10.1371/journal.pone.0230793 PMC7117706 32240231

[jonm13635-bib-0098] Pariona‐Cabrera, P. , Cavanagh, J. , & Bartram, T. (2020). Workplace violence against nurses in health care and the role of human resource management: A systematic review of the literature. Journal of Advanced Nursing, 76(7), 1581–1593. 10.1111/jan.14352 32175613

[jonm13635-bib-0099] Park, S.‐H. , & Choi, E.‐H. (2020). The cycle of verbal violence among nurse colleagues in South Korea. Journal of Interpersonal Violence, 37, NP3107–NP3129. 10.1177/0886260520945680 32772624

[jonm13635-bib-0100] Park, M. , Cho, S.‐H. , & Hong, H.‐J. (2015). Prevalence and perpetrators of workplace violence by nursing unit and the relationship between violence and the perceived work environment. Journal of Nursing Scholarship, 47(1), 87–95. 10.1111/jnu.12112 25352254

[jonm13635-bib-0101] Peters, M. D. J. , Godfrey, C. M. , Khalil, H. , McInerney, P. , Parker, D. , & Soares, C. B. (2015). Guidance for conducting systematic scoping reviews. International Journal of Evidence‐Based Healthcare, 13(3), 141–146. 10.1097/xeb.0000000000000050 26134548

[jonm13635-bib-0102] Peters, A. , El‐Ghaziri, M. , Quinn, B. , Simons, S. , & Taylor, R. (2020). An exploratory study of bullying exposure among school nurses: Prevalence and impact. The Journal of School Nursing, 37(6), 449–459. 10.1177/1059840519897308 31910730

[jonm13635-bib-0103] Peters, M. , Godfrey, C. , McInerney, P. , Munn, Z. , Trico, A. , & Khalil, H. (2020). Chapter 11: Scoping Reviews. In JBI manual for evidence synthesis. 10.46658/jbimes-20-12

[jonm13635-bib-0104] Pich, J. , & Roche, M. (2020). Violence on the job: The experiences of nurses and midwives with violence from patients and their friends and relatives. Healthcare (Basel), 8(4). 10.3390/healthcare8040522 PMC7712129 33266225

[jonm13635-bib-0105] Pinar, R. , & Ucmak, F. (2011). Verbal and physical violence in emergency departments: A survey of nurses in Istanbul, Turkey. Journal of Clinical Nursing, 20(3–4), 510–517. 10.1111/j.1365-2702.2010.03520.x 20969652

[jonm13635-bib-0106] Ramacciati, N. , Gili, A. , Mezzetti, A. , Ceccagnoli, A. , Addey, B. , & Rasero, L. (2019). Violence towards emergency nurses: The 2016 Italian National Survey—A cross‐sectional study. Journal of Nursing Management, 27(4), 792–805. 10.1111/jonm.12733 30430675

[jonm13635-bib-0107] Ramacciati, N. , Guazzini, A. , Caldelli, R. , & Rasero, L. (2021). User‐friendly system (a smartphone app) for reporting violent incidents in the emergency department: An Italian multicenter study. La Medicina del Lavoro, 112(1), 68–81. 10.23749/mdl.v112i1.9984 33635296 PMC8023056

[jonm13635-bib-0141] Ray, C. L. , & Subich, L. M. (1998). Staff assaults and injuries in a psychiatric hospital as a function of three attitudinal variables. Issues in Mental Health Nursing, 19(3), 277–289. 10.1080/016128498249079 9661378

[jonm13635-bib-0108] Read, E. , & Laschinger, H. K. (2013). Correlates of new graduate nurses' experiences of workplace mistreatment. The Journal of Nursing Administration, 43(4), 221–228. 10.1097/NNA.0b013e3182895a90 23528688

[jonm13635-bib-0109] Reknes, I. , Pallesen, S. , Magerøy, N. , Moen, B. E. , Bjorvatn, B. , & Einarsen, S. (2014). Exposure to bullying behaviors as a predictor of mental health problems among Norwegian nurses: Results from the prospective SUSSH‐survey. International Journal of Nursing Studies, 51(3), 479–487. 10.1016/j.ijnurstu.2013.06.017 23891534

[jonm13635-bib-0110] Rodney, V. (2000). Nurse stress associated with aggression in people with dementia: Its relationship to hardiness, cognitive appraisal and coping. Journal of Advanced Nursing, 31(1), 172–180. 10.1046/j.1365-2648.2000.01247.x 10632806

[jonm13635-bib-0111] Sakellaropoulos, A. , Pires, J. , Estes, D. , & Jasinski, D. (2011). Workplace aggression: Assessment of prevalence in the field of nurse anesthesia. AANA Journal, 79(4 Suppl), S51–S57. https://www.ncbi.nlm.nih.gov/pubmed/22403967 22403967

[jonm13635-bib-0112] Schlak, A. E. , Aiken, L. H. , Chittams, J. , Poghosyan, L. , & McHugh, M. (2021). Leveraging the work environment to minimize the negative impact of nurse burnout on patient outcomes. International Journal of Environmental Research and Public Health, 18(2). 10.3390/ijerph18020610 PMC7828279 33445764

[jonm13635-bib-0113] Seibel, L. M. , & Fehr, F. C. (2018). “They can crush you”: Nursing students experiences of bullying and the role of faculty. Journal of Nursing Education and Practice, 8(6), 66. 10.5430/jnep.v8n6p66

[jonm13635-bib-0114] Serafin, L. I. , & Czarkowska‐Pączek, B. (2019). Prevalence of bullying in the nursing workplace and determinant factors: A nationwide cross‐sectional polish study survey. BMJ Open, 9(12), e033819. 10.1136/bmjopen-2019-033819 PMC7008430 31801744

[jonm13635-bib-0115] Shi, L. , Zhang, D. , Zhou, C. , Yang, L. , Sun, T. , Hao, T. , Peng, X. , Gao, L. , Liu, W. , Mu, Y. , Han, Y. , & Fan, L. (2017). A cross–sectional study on the prevalence and associated risk factors for workplace violence against Chinese nurses. BMJ Open, 7(6), e013105. 10.1136/bmjopen-2016-013105 PMC5623406 28647719

[jonm13635-bib-0116] Soilemezi, D. , & Linceviciute, S. (2018). Synthesizing qualitative research. International Journal of Qualitative Methods, 17(1). 10.1177/1609406918768014

[jonm13635-bib-0117] Somani, R. , Muntaner, C. , Hillan, E. , Velonis, A. J. , & Smith, P. (2021). A systematic review: Effectiveness of interventions to De‐escalate workplace violence against nurses in healthcare settings. Safety and Health at Work, 12(3), 289–295. 10.1016/j.shaw.2021.04.004 34527388 PMC8430427

[jonm13635-bib-0118] Spelten, E. , Thomas, B. , O'Meara, P. , van Vuuren, J. , & McGillion, A. (2020). Violence against emergency department nurses; can we identify the perpetrators? PLoS ONE, 15(4), e0230793. 10.1371/journal.pone.0230793 32240231 PMC7117706

[jonm13635-bib-0119] Speroni, K. G. , Fitch, T. , Dawson, E. , Dugan, L. , & Atherton, M. (2014). Incidence and cost of nurse workplace violence perpetrated by hospital patients or patient visitors. Journal of Emergency Nursing, 40(3), 218–228. 10.1016/j.jen.2013.05.014 24054728

[jonm13635-bib-0120] Tee, S. , Üzar Özçetin, Y. S. , & Russell‐Westhead, M. (2016). Workplace violence experienced by nursing students: A UK survey. Nurse Education Today, 41, 30–35. 10.1016/j.nedt.2016.03.014 27138479

[jonm13635-bib-0121] Tomagová, M. , Zeleníková, R. , Kozáková, R. , Žiaková, K. , Babiarczyk, B. , & Turbiarz, A. (2020). Violence against nurses in healthcare facilities in the Czech Republic and Slovakia. Central European Journal of Nursing and Midwifery, 11(2), 52–61. 10.15452/cejnm.2020.11.0009

[jonm13635-bib-0122] Touzet, S. , Occelli, P. , Denis, A. , Cornut, P.‐L. , Fassier, J.‐B. , Le Pogam, M.‐A. , Duclos, A. , & Burillon, C. (2019). Impact of a comprehensive prevention programme aimed at reducing incivility and verbal violence against healthcare workers in a French ophthalmic emergency department: An interrupted time‐series study. BMJ Open, 9(9), e031054. 10.1136/bmjopen-2019-031054 PMC6731840 31492791

[jonm13635-bib-0123] Tricco, A. C. , Lillie, E. , Zarin, W. , O'Brien, K. K. , Colquhoun, H. , Levac, D. , … Straus, S. E. (2018). PRISMA Extension for Scoping Reviews (PRISMA‐ScR): Checklist and explanation. Annals of Internal Medicine, 169(7), 467–473. 10.7326/m18-0850 30178033

[jonm13635-bib-0124] Van Bogaert, P. , Peremans, L. , Van Heusden, D. , Verspuy, M. , Kureckova, V. , Van de Cruys, Z. , & Franck, E. (2017). Predictors of burnout, work engagement and nurse reported job outcomes and quality of care: A mixed method study. BMC Nursing, 16, 5. 10.1186/s12912-016-0200-4 28115912 PMC5241948

[jonm13635-bib-0125] Velden, P. G. , Bosmans, M. W. G. , & Meulen, E. (2015). Predictors of workplace violence among ambulance personnel: A longitudinal study. Nursing Open, 3(2), 90–98. 10.1002/nop2.38 27708819 PMC5047336

[jonm13635-bib-0126] Ventura‐Madangeng, J. , & Wilson, D. (2009). Workplace violence experienced by registered nurses: A concept analysis. Nursing Praxis in New Zealand, 25(3), 37–50. https://www.ncbi.nlm.nih.gov/pubmed/20157959 20157959

[jonm13635-bib-0127] Vincent‐Höper, S. , Stein, M. , Nienhaus, A. , & Schablon, A. (2020). Workplace aggression and burnout in nursing—The moderating role of follow‐up counseling. International Journal of Environmental Research and Public Health, 17(9). 10.3390/ijerph17093152 PMC7246829 32369903

[jonm13635-bib-0128] Wax, J. R. , Pinette, M. G. , & Cartin, A. (2016). Workplace violence in health care—Its not “part of the job”. Obstetrical & Gynecological Survey, 71(7), 427–434. 10.1097/ogx.0000000000000334 27436177

[jonm13635-bib-0129] Williams, M. F. (1996). Violence and sexual harassment: Impact on registered nurses in the workplace. AAOHN Journal, 44(2), 73–77. https://www.ncbi.nlm.nih.gov/pubmed/8694978, 10.1177/216507999604400204 8694978

[jonm13635-bib-0143] Wolf, L. A. , Perhats, C. , Delao, A. M. , & Clark, P. R. (2017). Workplace aggression as cause and effect: Emergency nurses’ experiences of working fatigued. International Emergency Nursing, 33, 48–52. 10.1016/j.ienj.2016.10.006 27919622

[jonm13635-bib-0130] Wu, Y. , Wang, J. , Liu, J. , Zheng, J. , Liu, K. , Baggs, J. G. , Liu, X. , & You, L. (2020). The impact of work environment on workplace violence, burnout and work attitudes for hospital nurses: A structural equation modelling analysis. Journal of Nursing Management, 28(3), 495–503. 10.1111/jonm.12947 31891429

[jonm13635-bib-0131] Xing, K. , Jiao, M. , Ma, H. , Qiao, H. , Hao, Y. , Li, Y. , Gao, L. , Sun, H. , Kang, Z. , Liang, L. , & Wu, Q. (2015). Physical violence against general practitioners and nurses in Chinese township hospitals: A cross‐sectional survey. PLoS ONE, 10(11), e0142954. 10.1371/journal.pone.0142954 26571388 PMC4646672

[jonm13635-bib-0132] Yang, L. Q. , Spector, P. E. , Chang, C. H. , Gallant‐Roman, M. , & Powell, J. (2012). Psychosocial precursors and physical consequences of workplace violence towards nurses: A longitudinal examination with naturally occurring groups in hospital settings. International Journal of Nursing Studies, 49(9), 1091–1102. 10.1016/j.ijnurstu.2012.03.006 22546849

[jonm13635-bib-0133] Yang, B. X. , Stone, T. E. , Petrini, M. A. , & Morris, D. L. (2018). Incidence, type, related factors, and effect of workplace violence on mental health nurses: A cross‐sectional survey. Archives of Psychiatric Nursing, 32(1), 31–38. 10.1016/j.apnu.2017.09.013 29413069

[jonm13635-bib-0134] Yokoyama, M. , Suzuki, M. , Takai, Y. , Igarashi, A. , Noguchi‐Watanabe, M. , & Yamamoto‐Mitani, N. (2016). Workplace bullying among nurses and their related factors in Japan: A cross‐sectional survey. Journal of Clinical Nursing, 25(17–18), 2478–2488. 10.1111/jocn.13270 27383562

[jonm13635-bib-0135] Zeng, J. Y. , An, F. R. , Xiang, Y. T. , Qi, Y. K. , Ungvari, G. S. , Newhouse, R. , Yu, D. S. , Lai, K. Y. , Yu, L. Y. , Ding, Y. M. , Tang, W. K. , Wu, P. P. , Hou, Z. J. , & Chiu, H. F. (2013). Frequency and risk factors of workplace violence on psychiatric nurses and its impact on their quality of life in China. Psychiatry Research, 210(2), 510–514. 10.1016/j.psychres.2013.06.013 23850435

[jonm13635-bib-0136] Zhu, H. , Liu, X. , Yao, L. , Zhou, L. , Qin, J. , Zhu, C. , Ye, Z. , & Pan, H. (2021). Workplace violence in primary hospitals and associated risk factors: A cross‐sectional study. Nursing Open, 9(1), 513–518. 10.1002/nop2.1090 34655279 PMC8685843

